# Hollow gold nanoshells-incorporated injectable genetically engineered hydrogel for sustained chemo-photothermal therapy of tumor

**DOI:** 10.1186/s12951-019-0532-9

**Published:** 2019-09-17

**Authors:** RuiMei Jin, Jie Yang, DongHui Zhao, XiaoLin Hou, ChaoQing Li, Wei Chen, YuanDi Zhao, ZhongYuan Yin, Bo Liu

**Affiliations:** 1 Britton Chance Center for Biomedical Photonics at Wuhan National Laboratory for Optoelectronics-Hubei Bioinformatics & Molecular Imaging Key Laboratory, Collaborative Innovation Center for Biomedical Engineering, College of Life Science and Technology, Huazhong University of Science and Technology, Wuhan, 430074 Hubei People’s Republic of China; 2 Key Laboratory of Biomedical Photonics (HUST), Ministry of Education, Huazhong University of Science and Technology, Wuhan, 430074 Hubei People’s Republic of China; 3 Cancer Center, Union Hospital, Tongji Medical College, Huazhong University of Science and Technology, Wuhan, 430022 Hubei People’s Republic of China

**Keywords:** Injectable hydrogel, Hollow gold nanoshells, Genetically engineered polypeptide, Chemotherapy, Photothermal therapy

## Abstract

**Background:**

Combined therapy has demonstrated to be an effective strategy for cancer therapy. Herein, an injectable hydrogel based on the genetically engineered polypeptide and hollow gold nanoshells (HAuNS) has been developed for chemo-photothermal therapy of HepG2 tumor.

**Methods:**

PC_10_A/DOX/HAuNS nanogel was prepared with layer-by-layer through the adsorption of DOX and PC_10_A successively. DOX with positive charge and PC_10_A with negative charge were coated step by step onto the surface of negatively charged HAuNS. The multifunctional hydrogel PC_10_A/DOX/HAuNS were prepared via dissolving hybrid PC_10_A/DOX/HAuNS nanogel in polypeptide PC_10_A. Chemotherapy drug DOX in the PC_10_A/DOX/HAuNS hydrogel was absorbed on the HAuNS and directly embedded in the PC_10_A hydrogel, which contributes to sequentially release of the drug. Specifically, DOX adsorbed on the HAuNS could be released slowly for sustainable chemotherapy.

**Results:**

The PC_10_A/DOX/HAuNS hydrogel could pass 26-gauge needle without clogging, indicating that it is injectable. In addition, the PC_10_A/DOX/HAuNS hydrogel possessed outstanding photothermal effect and photothermal stability. In both in vitro cell and in vivo tumor-bearing mice experiments, a remarkably enhance tumor inhibition was observed by the combined therapy of chemo-photothermal therapy compared with photothermal therapy or chemotherapy alone.

**Conclusions:**

The combined chemotherapy and photothermal therapy of PC_10_A/DOX/HAuNS hydrogels could significantly improve the therapeutic effect. Therefore, the multifunctional hydrogel PC_10_A/DOX/HAuNS is promising to provide a new strategy for sustained chemo-photothermal therapy.

## Introduction

Photothermal therapy (PTT), especially caused by near-infrared (NIR) light, has attracted lots of interest in tumor therapy owing to its deep tissue penetrability, non-radiative conversion of light energy, and specific spatial/temporal selectivity [1–3]. Various inorganic/organic nanomaterials, such as gold nanorod, hollow gold nanoshells (HAuNS), carbon tube, and indocyanine green were utilized as photothermal agents for PPT of tumors [4–7]. They are endowed with excellent light-to-heat conversion efficiency. For example, Chen et al. used MoSe_2_ nanosheet for effectively CT26 colorectal tumor therapy [8]. Among them, HAuNS was most widely used due to its lack of need for cytotoxic surfactant, tremendous drug load capacity, and excellent photothermal efficiency [9–12]. However, PTT alone could not ablate completely the tumor cells due to residual tumor mass at the treatment margins, which easily result in tumor recurrence [13]. Therefore, combination strategies are expected to improve the overall efficacy of tumor treatment.

Chemotherapy is the traditional malignant tumor cure method in clinical application, while high dose chemotherapeutic drug administration was usually implemented to maintain the therapeutic drug concentration at the expense of serious side effects to normal tissue cells [14–16]. Since the last decade, anticancer drugs have been incorporated easily into the hydrogel for local chemotherapy, which avoided the long journey in the circulatory system, decreased toxicity to normal tissues [17–21]. Especially, injectable hydrogels can be used as drug carriers to provide drug directly located at the desired position in high-concentrations and leading to improved utilization efficiency [22–24]. Days or even weeks of continuous drug release of injectable hydrogel can avoid the side effects of multiple administration. In addition, different functional anticancer drugs and functional nanomaterials can be loaded in the injectable hydrogel by a simple mixture for sequential release and combined therapy [25, 26]. To date, injectable hydrogels are mostly synthetic polymer or natural proteins [27]. However, the potential toxicity of synthetic polymer and the complex composition of natural proteins limit their materials as drug delivery carriers [28]. Genetically engineered polypeptides with single component and high biocompatibility are very favorable to prepare injectable hydrogels as drug delivery carriers [29]. Previous studies have shown that HAuNS were widely used in the combined treatment of tumors due to their strong photothermal effect and high drug loading capacity [30]. However, the clinical applications of HAuNS alone for tumor therapy still face many challenges due to drug leakage and unstability under physiological conditions [31]. To overcome these limitations, the strategy with combination of HAuNS and polypeptide hydrogel is expected to achieve a satisfactory treatment outcome for tumor therapy.

**Scheme 1:**
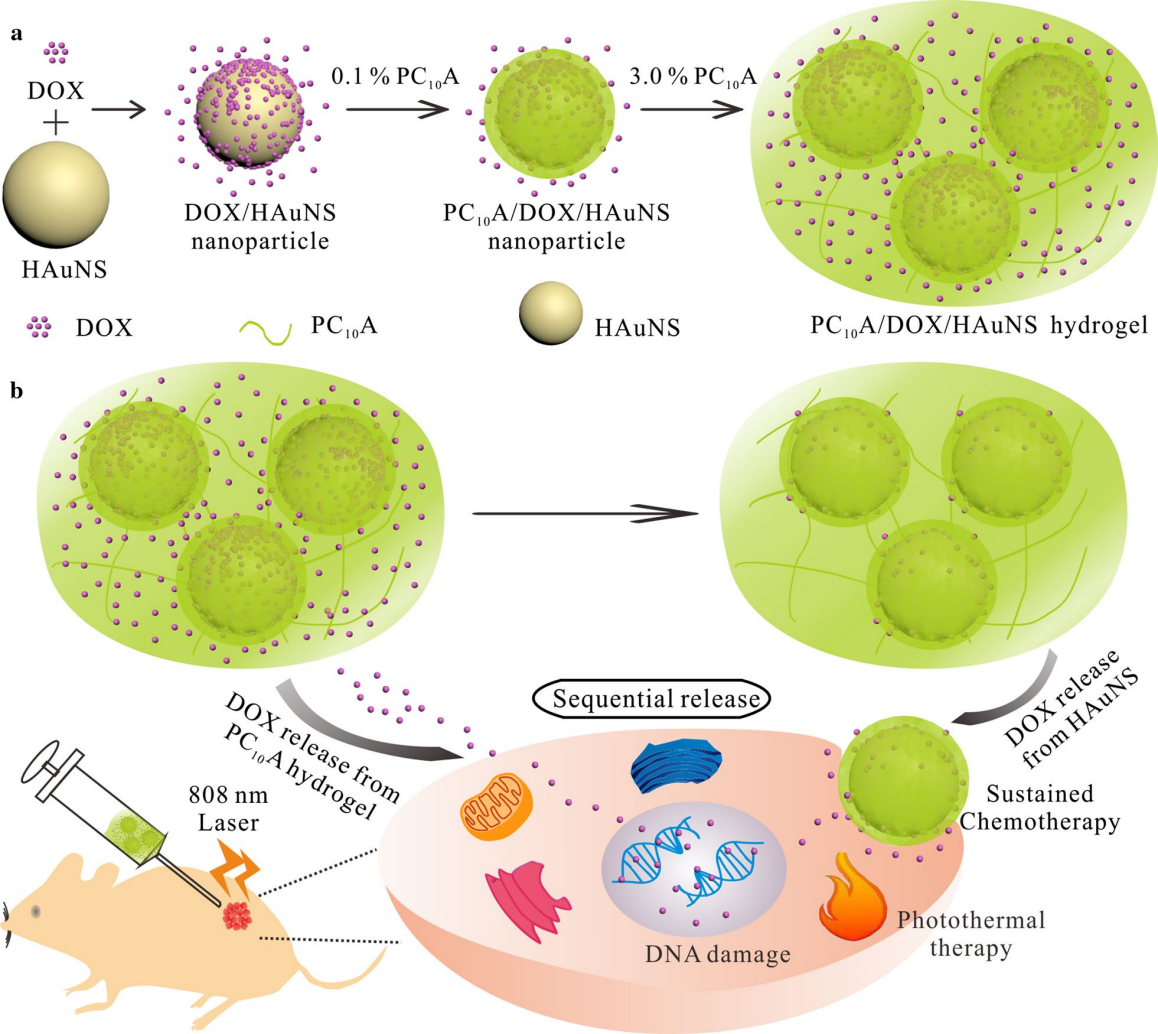
**a** Schematic illustration of the preparation of PC_10_A/DOX/HAuNS nanoparticles and PC_10_A/DOX/HAuNS hydrogel through self-assemble layer-by-layer and “dissolving” the PC_10_A/DOX/HAuNS nanoparticles in the PC_10_A hydrogel, respectively; **b** PC_10_A/DOX/HAuNS hydrogel with sequential drug release were utilized for sustained chemotherapy and photothermal therapy in vivo

Herein, we developed an injectable hydrogel based on the genetically engineered polypeptide and HAuNS for chemo-photothermal therapy of HepG2 tumor. We first prepared a drug-loaded hybrid PC_10_A/DOX/HAuNS nanogel through layer-by-layer coating with DOX and PC_10_A. The hybrid PC_10_A/DOX/HAuNS nanogels were dissolved in PC_10_A hydrogel to product injectable PC_10_A/DOX/HAuNS hydrogel exhibiting a variety of excellent functions (Scheme 1). One part of the drug DOX in the PC_10_A/DOX/HAuNS hydrogel was absorbed on the HAuNS, and the other part was directly embedded in the PC_10_A hydrogel, which contributes to sequentially release of the drug. The PC_10_A/DOX/HAuNS hydrogel had outstanding photothermal effect and photothermal stability. The results of toxicity showed that the PC_10_A/DOX/HAuNS hydrogel presented excellent biocompatibility. The results of in vivo experiments have demonstrated that the combination of photothermal therapy and chemotherapy of PC_10_A/DOX/HAuNS hydrogel exhibited a good inhibitory effect on HepG2 tumor, and the recurrence was controlled at a low level.

## Materials and methods

### Materials

Restriction endonuclease *Bam*HI, *Nhe*I, *Spe*I, and T4 DNA ligase were obtained from New England Biolabs Inc. (Beijing, China). Ampicillin and kanamycin were purchased from Sinopharm Group Chemical Reagent Co., Ltd (Shanghai, China). Isopropyl-*β*-d-thiogalactoside (IPTG) and nickelnitrilotriacetic acid (Ni-NTA) separation column were purchased from Qiagen Co., Ltd (Shanghai, China). Cobalt chloride hexahydrate (99.99%), gold chloride trihydrate (HAuCl_4_·3H_2_O), trisodium citrate dehydrate (> 99%), polyvinylpyrrolidone (PVP, MW: 55,000), sodium borohydride (99%), calcein AM, Propidium Iodide (PI), and Doxorubicin Hydrochloride (DOX) were obtained from Sigma-Aldrich, Inc. (USA). Ultrapure water (18.2 MΩ) purified by the Milli-Q system (Millipore, DE) was used to prepare all solutions.

### Preparation of the genetically engineered polypeptide PC_10_A, HAuNS nanoparticles, and PC_10_A/DOX/HAuNS hydrogels

PQE9PC_10_A plasmid was a gift from Prof. David Tirrell at the California Institute of Technology, Pasadena, CA. The genetically engineered polypeptide PC_10_A was prepared according to our previously reported method [32, 33]. The purified polypeptides were analyzed on a Bruker Reflex III reflectron MALDI-TOF mass spectrometer (Bremen, Germany).

HAuNS nanoparticles were synthesized by the replacement of cobalt nanoparticles in the presence of chloroauric acid according to the method of Schwartzberg et al. [34]. Briefly, 0.1 mM CoCl_2_·6H_2_O, 0.1 mM C_6_H_5_Na_3_O_7_·2H_2_O, and 10 μM PVP were dissolved in 100 mL water, stirred and protected with argon for 45 min. Eight hundred milliliters of 1 mM fresh sodium borohydride was added into the solution. The mixture was stirred for 60 min, and 430 μL HAuCl_4_·3H_2_O (1 g 100 mL^−1^) was added into the mixture dropwise. The mixture was stirred for 25 min and exposed in the air until it turned to green. The mixture was centrifuged at 14,000 rpm for 20 min and washed with ultrapure water for three times. Five-hundred microliters of DOX solution (1.6 mg mL^−1^) was dispersed into equal volume HAuNS nanoparticles solution (40 μg mL^−1^). After stirring for 24 h, the mixture was repeated centrifugation (10,000 rpm, 20 min) to remove free DOX and obtain DOX-loaded HAuNS/DOX nanoparticle.

The quantity of unloaded DOX in the supernatant was estimated by the absorbance at 480 nm. The drug loading rate was calculated as follows: Drug Loading Rate = (mass of total DOX − mass of DOX in the supernatant)/mass of HAuNS/DOX × 100%.

One milligram PC_10_A was dissolved into above mixture to prepare PC_10_A/DOX/HAuNS nanoparticles. Sizes of HAuNS nanoparticles, HAuNS/DOX nanoparticles and PC_10_A/DOX/HAuNS nanoparticles were measured by a Tecnai G2 20 U-Twin TEM. Zeta potentials, hydrodynamic sizes, and UV–vis spectra of these nanoparticles were tested by a ZS90 electronic dynamic light scattering apparatus nanosizer (Malvern, U.K.) and a UV–vis spectrometer (Shimadzu, Japan), respectively.

Another 29 mg PC_10_A was dissolved in the PC_10_A/DOX/HAuNS solution, and pH of the solution was adjusted to 7.4 to prepare the PC_10_A/DOX/HAuNS hydrogel (PC_10_A: 3% w/w, DOX: 0.8 mg mL^−1^, HAuNS: 20 μg mL^−1^). The PC_10_A hydrogel, PC_10_A/DOX hydrogel, and PC_10_A/HAuNS hydrogel were prepared by the same method. The morphology of lyophilized PC_10_A hydrogel, PC_10_A/DOX hydrogel, PC_10_A/HAuNS hydrogel, and PC_10_A/HAuNS/DOX hydrogel was measured on a Nova Nano SEM 450 (FEI, USA).

### Rheological tests of PC_10_A hydrogel and PC_10_A/HAuNS/DOX hydrogel

The rheology properties of PC_10_A/DOX/HAuNS hydrogel and blank PC_10_A hydrogel were carried out on a HR-2 hybrid rheometer (TA Instrument). PC_10_A (PC_10_A: 3% w/w) and PC_10_A/DOX/HAuNS hydrogel (PC_10_A: 3% w/w, DOX: 0.8 mg mL^−1^, HAuNS: 20 μg mL^−1^) samples were mildly transferred into the middle of the 15 mm diameter parallel plate with a proper gap. Dynamic oscillatory frequency sweep measurements were conducted at the strain between 1% and 500%. Angular frequency dependent oscillatory rheology was tested from 10^−2^ to 10^3^ rad s^−1^. Self-healing ability was measured by repeating dynamic strain of 1% and 500%. Viscosity test was operated at the shear rate between 1 and 10 s^−1^. All the measurements were repeated for three times.

### Photothermal effect of PC_10_A/DOX/HAuNS hydrogel

Different concentrations of HAuNS nanoparticles (5, 10, 15, 20, 25 μg mL^−1^) were exposed with an 808 nm (2.0 W cm^−2^) laser for 9 min, and the temperatures were recorded by an EasIR-9 Thermal Imager (Wuhan Guide Infrared Co., Ltd, China). In addition, HAuNS solution (20 μg mL^−1^) were irradiated with an 808 nm laser with different power densities (0.5, 1, 1.5, 2.0 and 2.5 W cm^−2^), and the temperatures were also measured. Each experiment was repeated three times. The photothermal stability of HAuNS solution was measured by 6 circles laser irradiation on/off (λ = 808 nm, 2.0 W cm^−2^). In addition, the photothermal effect of PC_10_A hydrogel, PC_10_A/HAuNS hydrogel and PC_10_A/DOX/HAuNS hydrogel were measured with the same method of HAuNS nanoparticles. The η value of HAuNS nanoparticles was calculated through previously reported method [35].

### Cytotoxicity and hemolysis percentage of PC_10_A/HAuNS hydrogel in vitro

HepG2 cells in DMEM (with 10% FBS) were seeded in 96-well plates (5000 cells per well) and cultured in a cell incubator (5% CO_2_, 37 °C). After incubation for 20 h, the cells were washed with PBS and cultured with different concentrations of PC_10_A nanogel (0.8%, 0.4%, 0.2%, 0.1%, 0.05%) and HAuNS (HAuNS: 40, 20, 10, 5, 2.5 μg mL^−1^) in serum-free DMEM for another 48 h. The variances of cells were measured through standard MTT assay.

The biocompatibility of PC_10_A hydrogels and PC_10_A/HAuNS hydrogels were also tested by 3D cell culture. HepG2 cells (2 × 10^4^ cells per milliliter hydrogel) were dispersed into PC_10_A hydrogel (PC_10_A: 3% w/w) and PC_10_A/HAuNS hydrogel (PC_10_A: 3% w/w, HAuNS: 20 μg mL^−1^) containing 100 units/mL penicillin and streptomycin, respectively. The cell-laden hydrogels were transferred into 35 mm glass bottom culture dishes and cultured at 37 °C under a humidified 5% CO_2_ for 24 and 48 h. The cells were stained with the calcein AM and PI for 20 min and imaged with a 20× objective on an Olympus FLUOVIEW FV1000 confocal microscope (OLYMPUS, Japan).

One microliter of RBC was added on the PC_10_A hydrogel (200 μL, 3% w/w) and PC_10_A/HAuNS hydrogel (200 μL; PC_10_A: 3% w/w, HAuNS: 20 μg mL^−1^), respectively. Equivalent PBS and water with RBC were set as controls. All of them were cultured at 37 °C for different times (2, 4, and 8 h). The mixture was centrifuged at 3500 rpm for 5 min, and the absorbance of supernatant was measured at 577 nm. The hemolysis ration was calculated by following equation: Hemolysis Ration = (OD_Sample_ − OD_PBS_)/(OD_Water_ − OD_PBS_)*100%, OD_Sample_, OD_PBS_, and OD_Water_ is the absorbance value of the corresponding supernatant at 577 nm.

### Degradation of PC_10_A/DOX/HAuNS hydrogel and DOX release

PC_10_A hydrogel (120 mg) was transferred into a cylindrical tube through centrifugation. PBS (3 mL) was added on the PC_10_A hydrogel, and the tube was incubated in 37 °C. The erosion profiles of PC_10_A hydrogel in the PBS were determined by measuring the absorbance at 278 nm at successive time points, and equal volume fresh PBS was replaced. In addition, DOX release from PC_10_A/DOX/HAuNS hydrogel and PC_10_A/DOX hydrogel was tested. The PC_10_A/DOX/HAuNS hydrogel (0.5 mL) contained concentrated DOX/HAuNS (HAuNS: 20 μg mL^−1^, DOX: 0.8 mg mL^−1^) and PC_10_A/DOX (DOX 0.8 mg mL^−1^) were prepared and transferred into cylindrical tubes, respectively. Equivalent volume of PBS buffer (pH 7.4) or acetate buffer (pH 5.0) was added on the hydrogels. At each time point, 0.5 mL buffer was taken out and replaced with an equivalent volume of fresh buffer. The release of DOX was detected by the measurement absorption spectra at 480 nm on a UV-2550 UV–vis spectrophotometer (Shimadzu, Japan).

### Intratumoral retention and drug distribution in different organs

PC_10_A/IR783 hydrogel (PC_10_A: 3% w/w, IR783: 40 μg mL^−1^) was prepared by the similar method described above. Two groups of HepG2 tumor-bearing mice were dividedly injected with 100 μL free IR783 solution and PC_10_A/IR783 hydrogel with the same dose of IR783 (40 μg mL^−1^) by intratumoral injection. The imaging experiments were performed using a home-built mouse image system equipped with an excitation band pass filter at 740 nm and an emission optical filter at 800 nm. On day 6, one mouse of each group sacrificed, the major organs and tumor were imaged on collected on the home-built fluorescence system.

### Combination of chemotherapy and phototherapy in vitro and in vivo

HepG2 cells (20,000 cells per milliliter hydrogel) were dividedly embedded in blank PC_10_A hydrogel, PC_10_A/HAuNS hydrogel, PC_10_A/DOX hydrogel, and PC_10_A/DOX/HAuNS hydrogel (PC_10_A: 3%, HAuNS: 20 μg mL^−1^, DOX: 0.8 mg mL^−1^) and cultured for 24 h. Cells in blank PC_10_A hydrogel, PC_10_A/HAuNS hydrogel hydrogel, and PC_10_A/DOX/HAuNS hydrogel were irradiated with an 808 nm laser at a power density of 2.0 W cm^−2^ for 9 min. Following, the cells were stained with calcein AM and PI and imaged with a 20× objective on an Olympus FLUOVIEW FV1000 confocal microscope (OLYMPUS, Japan).

In addition, the photothermal efficiency of PC_10_A/DOX/HAuNS hydrogel in vivo was tested. One hundred microliter PC_10_A/DOX/HAuNS hydrogel (PC_10_A: 3% w/w, HAuNS: 20 μg mL^−1^, DOX: 0.8 mg mL^−1^) and blank PC_10_A hydrogel (PC_10_A: 3%) were separately injected into two groups of HepG2 tumor-bearing mice by intratumoral injection. All the tumors were irradiated under an 808 nm laser at the density of 2.0 W cm^−2^ for 9 min, and the temperature changes were recorded by an EasIR-9 Thermal Imager.

Forty-two HepG2 tumors-bearing male mice with the average tumor size ranging from 100 to 150 mm^3^ were randomly separated to seven groups, namely: (1) PBS group, (2) laser alone group, (3) PC_10_A group, (4) Free DOX group, (5) PC_10_A/DOX hydrogel group, (6) PC_10_A/HAuNS (laser+) hydrogel group, and (7) PC_10_A/DOX/HAuNS (laser+) hydrogel group (n = 7). One hundred microliter PBS, PC_10_A hydrogel, PC_10_A/HAuNS hydrogel, free DOX solution, PC_10_A/DOX hydrogel, and PC_10_A/DOX/HAuNS hydrogel (PC_10_A: 3% w/v, HAuNS: 20 μg mL^−1^, DOX: 0.8 mg/mL) was injected into the tumor, respectively. Mice of the laser alone group, PC_10_A/HAuNS group, and PC_10_A/DOX/HAuNS group were treated with laser irradiation (808 nm, 2 W cm^−2^) for 9 min. One tumor of each group was harvested after treatment for 24 h to H&E stain. The tumor sizes and body weights were recorded during the cure process. At the end of the cure, all the mice were sacrificed for the major organs and blood samples used for H&E stain and blood analysis.

### Long-term tumor therapy efficiency and recurrence rate

Fifteen HepG2 tumor-bearing male mice were randomly divided into three groups (n = 5) to study long-term therapy efficiency and recurrence rate of PC_10_A/DOX/HAuNS hydrogel. Mice were injected with PC_10_A/DOX hydrogel, PC_10_A/HAuNS hydrogel, and PC_10_A/DOX/HAuNS hydrogel by intratumoral injection. Except for the PC_10_A/DOX group, all the other tumors were treated with laser irradiation (808 nm, 2 W cm^−2^) for 10 min. On day 72, one tumor in the PC_10_A/DOX/HAuNS hydrogels group was dissected to inspect the residual hydrogel. The cure course was continued for 72 days. Tumors were divided and made photograph at the end of the cure. Survival percent was recorded during this course.

## Results and discussion

### Characterization of multifunctional PC_10_A/DOX/HAuNS hydrogel

**Fig. 1:**
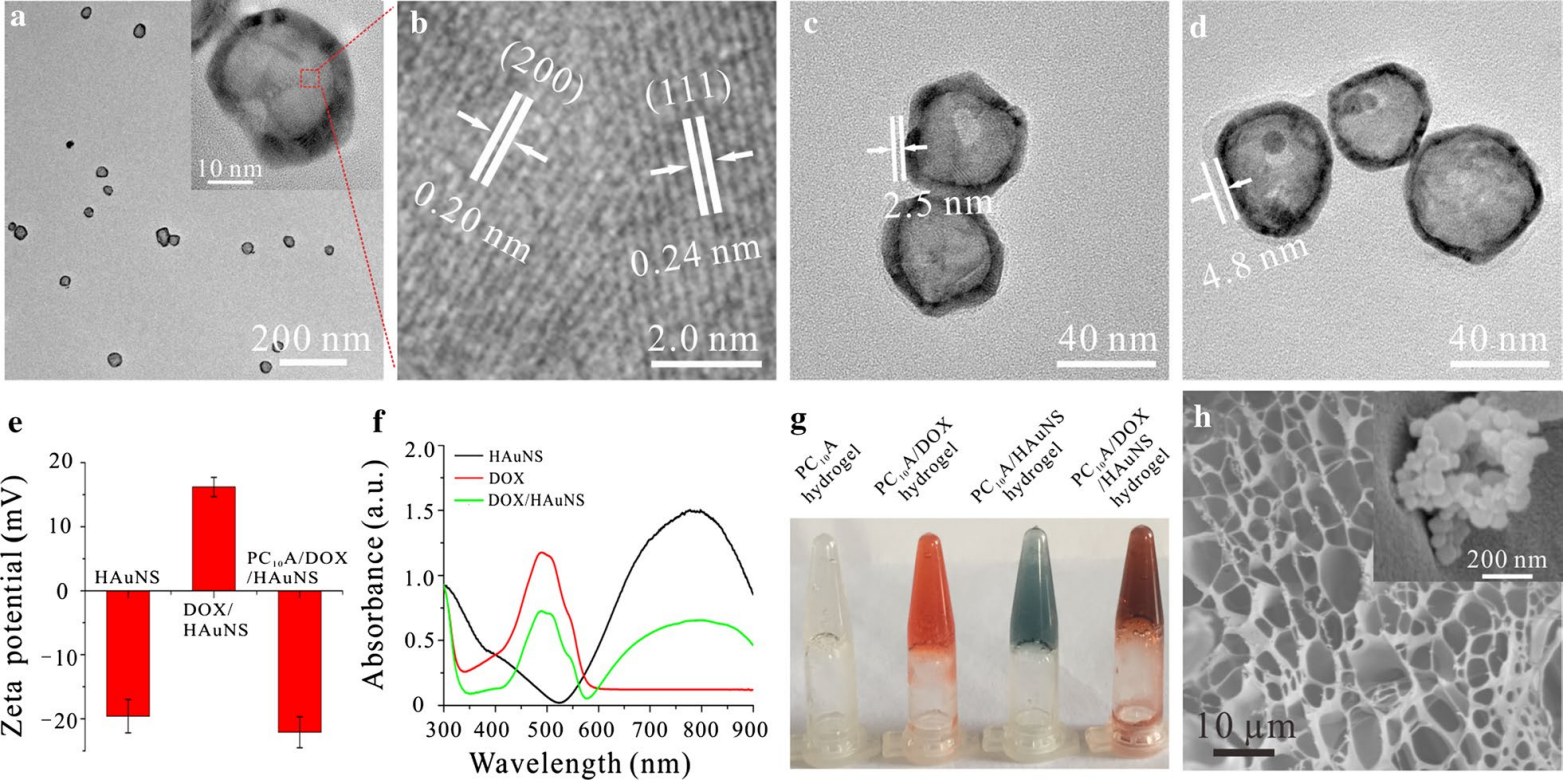
Characterization of PC_10_A/DOX/HAuNS nanogels and PC_10_A/DOX/HAuNS hydrogels. **a** TEM image and high resolution TEM image (insert) of HAuNS; **b** the different orientate lattice of HAuNS; TEM image of HAuNS/DOX nanoparticles (**c**) and PC_10_A/DOX/HAuNS nanoparticles (**d**); **e** zeta potentials of HAuNS, DOX/HAuNS nanoparticles, and PC_10_A/DOX/HAuNS nanoparticles; **f** UV–vis absorbance spectra of HAuNS solution, DOX solution, and DOX/HAuNS solution; **g** photographs of PC_10_A hydrogel, PC_10_A/DOX hydrogel, PC_10_A/HAuNS hydrogel, and PC_10_A/DOX/HAuNS hydrogel (PC_10_A: 3% w/w, DOX: 0.8 mg mL^−1^, HAuNS: 20 μg mL^−1^); **h** SEM image and the high resolution SEM (insert) of the PC_10_A/DOX/HAuNS hydrogel

A triblock genetically engineered polypeptide PC_10_A containing two coiled-coil domains P and A, which were located in the two terminals of the polypeptide, and a soluble random coil midblock C_10_ were prepared according to our previously reported method [32, 33]. The mass of the polypeptide PC_10_A was analyzed by a Bruker Reflex III reflectron MALDI-TOF mass spectrometer. PC_10_A (MS: 20,932.6 Da, the theoretical calculation of molecular weight: 20,858.5 Da). In addition, HAuNS with maximum absorption peak of 782 nm were synthesized through the method reported by Schwartzberg [34]. TEM images showed that HAuNS were homogenous with the diameter about 37.65 ± 4.23 nm (Fig. 1a). The different orientate lattice spaces with the gap about 0.20 nm and 0.24 nm agreed with the (111) and (200) crystal face, respectively, proving that HAuNS possessed the significant polycrystalline structure (Fig. 1b). To construct the multifunctional PC_10_A/DOX/HAuNS hydrogel, hybrid PC_10_A/DOX/HAuNS nanoparticles were prepared first. DOX with positive charge and PC_10_A with negative charge were coated step by step onto the surface of negatively charged HAuNS nanoparticles to prepare the hybrid PC_10_A/DOX/HAuNS nanoparticles. Compared to the HAuNS nanoparticles, an obviously DOX layer with thickness about 2.5 nm could be seen on the surface of DOX/HAuNS nanoparticles (Fig. 1c). In addition, the coating layer of the HAuNS nanoparticles after modifying with PC_10_A increased to 4.8 nm (Fig. 1d), which may attribute to the second coating of PC_10_A polypeptide on the out layer of DOX/HAuNS. The results of dynamic light scattering analysis showed that the hydrodynamic sizes of HAuNS, DOX/HAuNS, and PC_10_A/DOX/HAuNS increased gradually (Additional file 1: Fig. S1), which was consistent with those of TEM images. To further confirm that the hybrid PC_10_A/DOX/HAuNS nanoparticles were formed by electrostatic adsorption layer-by-layer, the zeta potentials of HAuNS, DOX/HAuNS, and PC_10_A/DOX/HAuNS were measured (Fig. 1e). The surface charges of DOX/HAuNS changed from negative (− 19.6 mV) to positive (+ 16.2 mV) because of positively charged DOX, indicating that DOX was successfully coated on the surface of HAuNS. While HAuNS coated with DOX followed by a layer of negatively charged PC_10_A exhibited negative charge (− 22.2 mV). These results demonstrated that hybrid PC_10_A/DOX/HAuNS nanoparticles were self-assembled layer-by-layer through the electrostatic adsorption. Compared with the absorption spectra of DOX and HAuNS, the characteristic absorption peak of DOX at 480 nm and the localized surface plasmon resonance peak of HAuNS at 782 nm were observed in the absorption spectrum of HAuNS/DOX (Fig. 1f), further indicating that DOX was successfully coated on the surface of HAuNS. Previous studies showed that PC_10_A could form stable physical hydrogel through self-assemble when its concentration was above 2% (w/w). Hybrid PC_10_A/DOX/HAuNS hydrogels were prepared through “dissolving” the PC_10_A/DOX/HAuNS nanoparticles in the PC_10_A hydrogel because they both contained the same composition PC_10_A (Fig. 1g). SEM image showed that the PC_10_A/DOX/HAuNS hydrogel was composed of a regular fiber framework and displayed highly porous interconnected structure (Fig. 1h). The HAuNS nanoparticle could be observed in the hydrogel in the high resolution SEM image (Fig. 1h, insert, Additional file 1: Fig. S2). No obvious difference of morphology was observed between blank PC_10_A hydrogel, PC_10_A/DOX hydrogel, PC_10_A/HAuNS hydrogel, and PC_10_A/DOX/HAuNS hydrogel (Additional file 1: Fig. S3), their diameters of the pore crossed by skeleton were about 10 ± 5 μm, indicating that the addition of DOX and HAuNS did not affect the structures of these hydrogels.

### Rheological behaviors of PC_10_A/DOX/HAuNS hydrogel

**Fig. 2:**
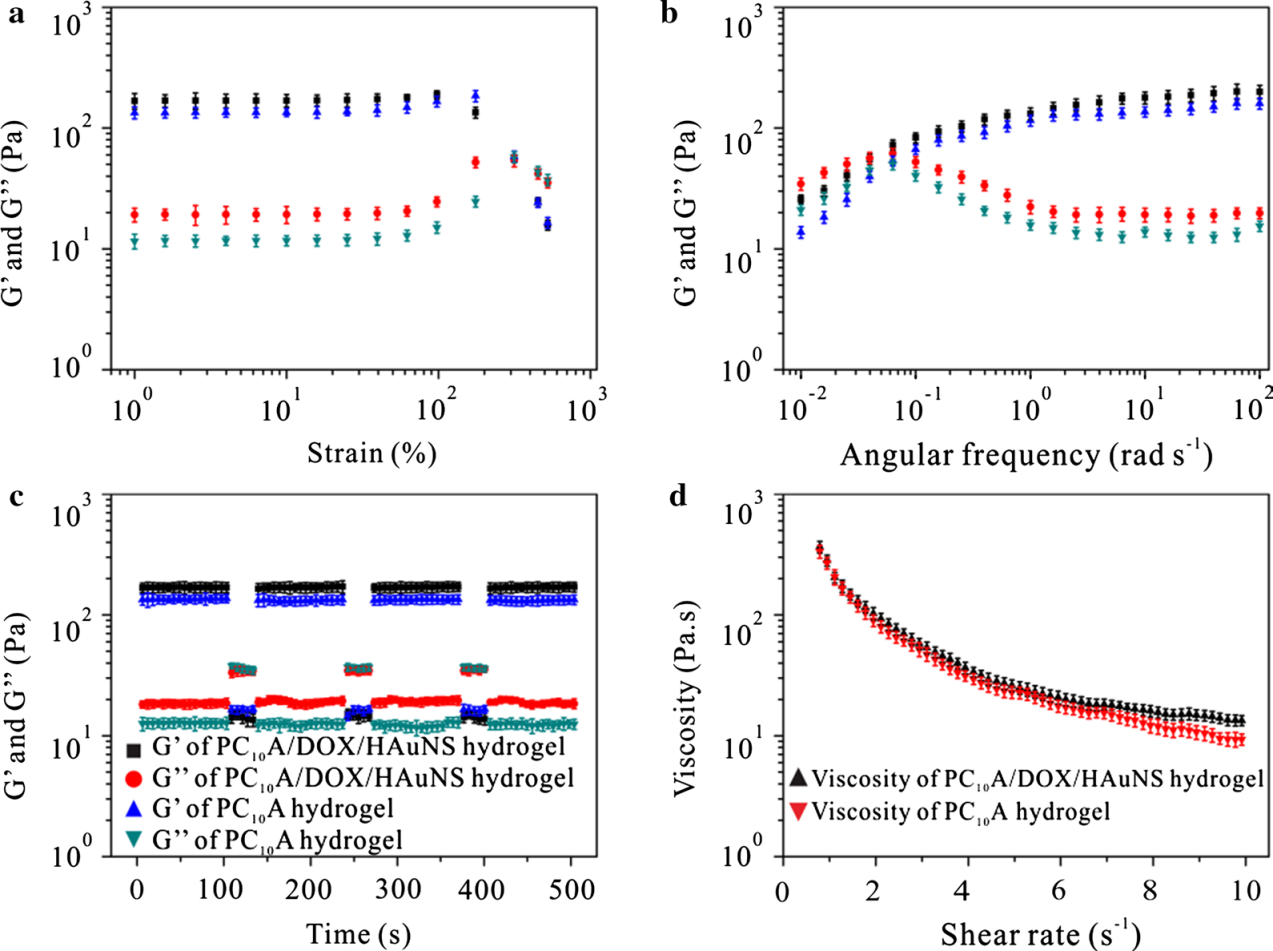
Rheological characterizations of PC_10_A hydrogels and PC_10_A/DOX/HAuNS hydrogels. **a** Dynamic oscillatory frequency sweep measurement (ω = 6.28 rad s^−1^) and **b** frequency dependent (strain = 1%) oscillatory shear rheology of the 3% w/w PC_10_A hydrogel and PC_10_A/DOX/HAuNS hydrogel (PC_10_A: 3% w/w, DOX: 0.8 mg mL^−1^, HAuNS: 20 μg mL^−1^); **c** the self-repairing properties of the PC_10_A hydrogel and PC_10_A/DOX/HAuNS hydrogel (PC_10_A: 3% w/w, DOX: 0.8 mg mL^−1^, HAuNS: 20 μg mL^−1^) demonstrated by the step-strain tests at an alternative strain of 1% and 500%; **d** viscosity curve of the 3% w/w PC_10_A hydrogel and PC_10_A/DOX/HAuNS hydrogel (PC_10_A: 3% w/w, DOX: 0.8 mg mL^−1^, HAuNS: 20 μg mL^−1^). n = 3

To characterize the viscoelastic properties of the PC_10_A/DOX/HAuNS hydrogel, rheology experiments of PC_10_A hydrogels and PC_10_A/DOX/HAuNS hydrogels were tested on a DHR-2 rheometer. Both hydrogels possessed similar viscoelastic features (Fig. 2a). Under the strain of 1–100%, storage moduli (G′) values of PC_10_A hydrogels and PC_10_A/DOX/HAuNS hydrogels were five times bigger than that of loss moduli (G″) at the angular frequency of 6.28 rad/s. This result indicated that both PC_10_A hydrogels and PC_10_A/DOX/HAuNS hydrogels exhibited linear viscoelastic behavior. With the further increase of strain, the sharp decrease of G′ and the gradual increase of G″ resulted in G′ < G″. When the strain was beyond 300%, PC_10_A hydrogels and PC_10_A/DOX/HAuNS hydrogels displayed nonlinear viscoelastic behavior. These results suggested that these hydrogels were physical hydrogels [36, 37]. Angular frequency dependent oscillatory rheology experiment was also performed (Fig. 2b). G′ increased along with the angular frequency from 0.01 to 100 rad s^−1^. While G″ trended to decrease after the angular frequency came to 0.06 rad/s. The cross point between G′ and G″ implied the phase transformation moment. When the HAuNS nanoparticles were incorporated into the PC_10_A hydrogel (3% w/w), the G′ of hydrogel increased from 134 to 178 Pa, indicating that the strength of PC_10_A hydrogel could be tuned by the addition of HAuNS nanoparticles. This result is probably due to the fact that HAuNS in the hydrogel can act as cross-linkers in the hydrogel. Continuous changing strain between 500% and 1% at the same frequency (6.28 rad s^−1^) was performed to assess the strain-induced damage and self-healing ability of these hydrogels (Fig. 2c). G′ and G″ remained stable after 3 cycles of breaking and reforming, supporting that these hydrogels possessed favourable self-healing properties. In addition, a continuous flow experiment with the shear rate ranging from 1 to 10 s^−1^ under the constant strain of 1% was operated (Fig. 2d). The viscosity decreased with the increase of shear rate, indicating that these hydrogels presented considerable shear-thinning property. This property is the basis for the injectable ability of hydrogels [38]. No obvious difference between the two viscosity curves of PC_10_A hydrogel and PC_10_A/DOX/HAuNS hydrogel was observed, suggesting that the addition of HAuNS nanoparticle and DOX had little effect on the viscosity of PC_10_A hydrogel. Excitingly, PC_10_A/DOX/HAuNS hybrid hydrogel could be transferred into the syringe and pass a 26-gauge needle without clogging (Additional file 1: Fig. S4), which was consistent with the result of viscosity test. Therefore, the PC_10_A/DOX/HAuNS hydrogel was expected to be used as an injectable carrier for tumor therapy.

### Photothermal effect of PC_10_A/DOX/HAuNS hydrogel

**Fig. 3:**
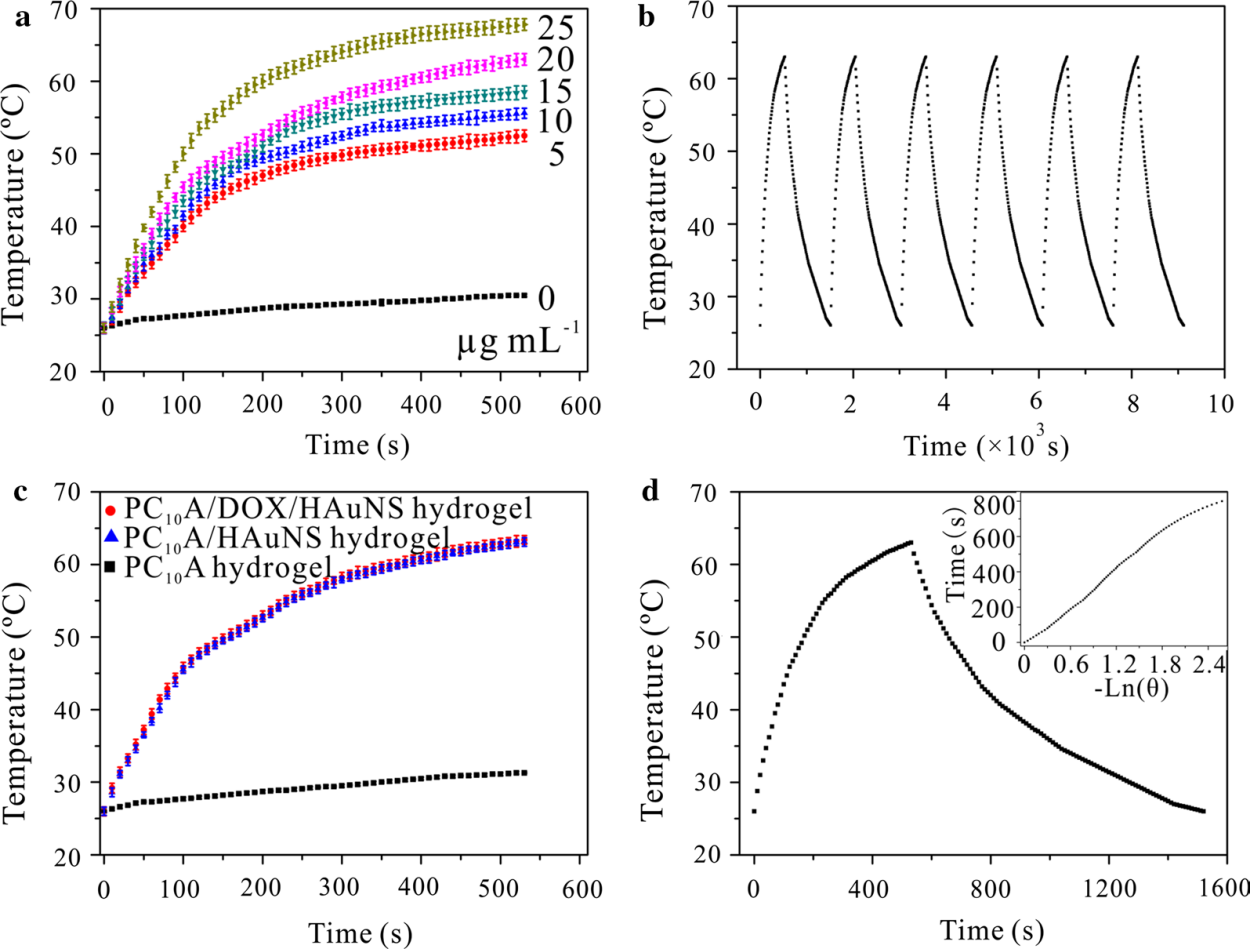
Photothermal characterizations of PC_10_A/DOX/HAuNS hydrogels. **a** Temperature changes of PC_10_A/DOX/HAuNS nanogels (PC_10_A: 0.1% w/w, DOX: 0.8 mg mL^−1^) containing different concentrations of HAuNS (25, 20, 15, 10, 5, and 0 μg mL^−1^) under irradiation with an 808 nm laser for 9 min (2.0 W cm^−2^); **b** photothermal stability of the HAuNS under six irradiation on/off cycles (808 nm, 2.0 W cm^−2^); **c** temperature changes of the PC_10_A hydrogel, PC_10_A/HAuNS hydrogel, and PC_10_A/DOX/HAuNS hydrogel (PC_10_A: 3% w/w, DOX: 0.8 mg mL^−1^, HAuNS: 20 μg mL^−1^) under irradiation (2.0 W cm^−2^) with an 808 nm laser for 9 min; **d** temperature changes of the PC_10_A/DOX/HAuNS nanogel (PC_10_A: 0.1% w/w, DOX: 0.8 mg mL^−1^, HAuNS: 20 μg mL^−1^) under laser irradiation (808 nm, 2.0 W cm^−2^) for 9 min and cooling for 16 min. n = 3

The photothermal conversion ability of photothermal agents plays an important role in photothermal therapy. To evaluate the photothermal effect of HAuNS nanoparticles in the PC_10_A/DOX/HAuNS hydrogel, different concentrations of HAuNS were exposed with an 808 nm laser (2.0 W cm^−2^). Temperatures of HAuNS solution increased quickly and eventually reached a plateau within 9 min (Fig. 3a), indicating that HAuNS can absorb the light and efficiently convert light energy to thermal energy. The photothermal effects of HAuNS solution under different laser power densities were also evaluated. The temperatures of HAuNS solution improved along with the increase of power density of the laser (Additional file 1: Fig. S5). These results exhibited that the temperature rising rates and the final temperature of the HAuNS solution under laser irradiation were depended on the concentration of HAuNS and the density of the laser.

Subsequently, photothermal stability of HAuNS nanoparticles was examined. HAuNS were exposed under the laser irradiation for 9 min and cooled it down at room temperature (Fig. 3b). After six cycles of laser irradiation, no obvious difference of the temperature change curve was observed. This result clarified that HAuNS nanoparticles presented outstanding photothermal stability. The photothermal effect of PC_10_A hydrogel, PC_10_A/HAuNS hydrogel, and PC_10_A/DOX/HAuNS hydrogel were also investigated. The temperature increasing trend of PC_10_A/HAuNS hydrogel and PC_10_A/DOX/HAuNS hydrogel was the same as those of HAuNS nanoparticles with the same concentration (Fig. 3a, c), while the increased temperatures of PC_10_A hydrogel were nearly negligible. These results indicated that HAuNS nanoparticles in the PC_10_A hysdrogel still emerged prominent photothermal effect. PC_10_A/HAuNS hydrogel and PC_10_A/DOX/HAuNS hydrogel trended to flow state after irradiation with a laser, while it returned to gel state after cooling (Additional file 1: Fig. S6). It is probably because PC_10_A in the hydrogel is a thermosensitive polypeptide. This result further demonstrated that PC_10_A hydrogel was a reversible physical hydrogel in response to changes in temperature. A typical temperature versus time plot of HAuNS was obtained with the irradiation of 2.0 W cm^−2^ for 9 min and cooling down under room temperature (Fig. 3d). The η value of HAuNS nanoparticle was measured by Roper’s method [35]. The value of η was 12.04%, indicating that HAuNS could use as an excellent photothermal agent for photothermal therapy of tumors.

### Biocompatibility and hemolysis ration of PC_10_A hydrogel in vitro

**Fig. 4:**
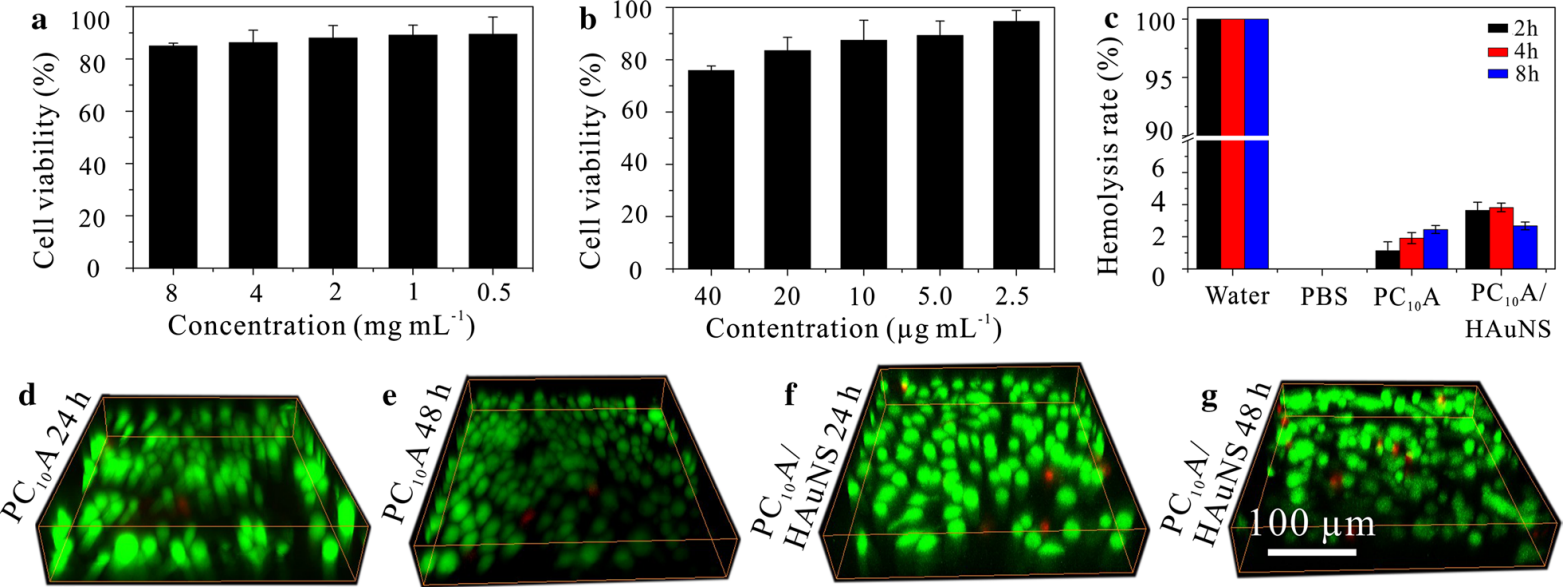
Biocompatibility of PC_10_A nanogels and hemolysis ration of PC_10_A hydrogel. The cell viability of HepG2 cells incubated with different concentrations of PC_10_A nanogels (**a**) and HAuNS (**b**); **c** hemolysis ration of PC_10_A hydrogel and PC_10_A/HAuNS hydrogel incubated with RBC for a series of time points (2, 4, and 8 h); **d**–**g** 3D cell culture of HepG2 cells in PC_10_A hydrogel (3% w/w) and PC_10_A/HAuNS hydrogel (PC_10_A: 3% w/w; HAuNS: 20 μg mL^−1^) for 24 h and 48 h

To assess the biocompatibility of PC_10_A/HAuNS hydrogels, the cell viabilities of HepG2 cells cultured with different concentrations of PC_10_A and HAuNS were tested by MTT assay. The cell viabilities of HepG2 cells were above 80% even the concentration of PC_10_A as high as 8 mg mL^−1^ or HAuNS as high as 20 μg mL^−1^ (Fig. 4a, b), which suggested that both PC_10_A and HAuNS were non-toxic to HepG2 cells. At the same time, the biocompatibility of PC_10_A hydrogel and PC_10_A/HAuNS hydrogel were evaluated through 3D culture of HepG2 cells. After 48 h incubation, the cell viability of HepG2 cells in hydrogels was above 90% (Fig. 4d–g). In addition, hemolysis ration experiments were operated though incubated red blood cell (RBC) with PC_10_A hydrogel and PC_10_A/HAuNS hydrogel (Fig. 4c). To serve as control groups, hemolysis ration experiments in ultrapure water and PBS were also investigated. The result showed that RBC in ultrapure water emerged completely hemolysis within 2 h. However, the hemolysis rates of PC_10_A hydrogel and PC_10_A/HAuNS hydrogel were below 4% after incubation for 8 h. This result further demonstrated that PC_10_A hydrogel was promised to be applied as a safety drug carrier for biomedical application.

### Hydrogel erosion and DOX release in vitro

**Fig. 5:**
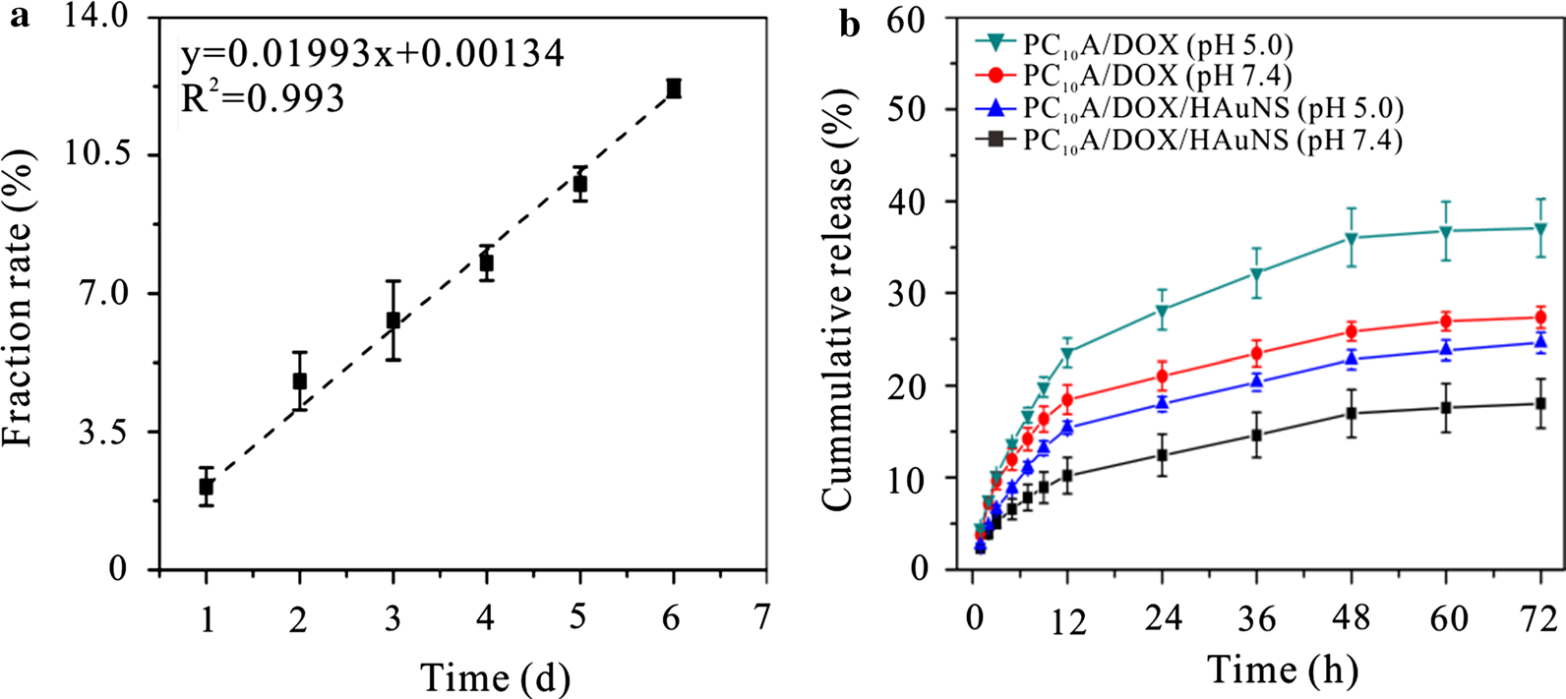
Fraction rate of PC_10_A hydrogel (3% w/w) (**a**) and cumulative release of DOX from PC_10_A/DOX hydrogel and PC_10_A/DOX/HAuNS hydrogel (**b**) (PC_10_A: 3% w/w; DOX: 0.8 mg mL^−1^; HAuNS: 20 μg mL^−1^) at different pH

PC_10_A hydrogel is a physical hydrogel formed by the coiled-coil P and A domains self-assembly, which can degrade slowly in PBS [39]. The erosion profile of 3% w/w PC_10_A hydrogel showed linear degradation behavior with time in the open aqueous solution system (Fig. 5a). At 37 °C in PBS, the accumulative release of a 3% w/w PC_10_A hydrogel was about 13% in 6 days. This result indicated that erosion was emerged on the surface rather than in whole bulk. In addition, the release of DOX from PC_10_A/DOX/HAuNS hydrogels at different pH was evaluated. As shown in Fig. 5b, DOX was released rapidly in the first 12 h, and the release rate of DOX decreased gradually after 12 h. Compared with the DOX released from PC_10_A/DOX/HAuNS hydrogels, the release rate and accumulative release of DOX from PC_10_A/DOX hydrogels were faster and greater, which may be due to the existence of two forms of DOX in PC_10_A/DOX/HAuNS hydrogels, one was directly embedded in the PC_10_A hydrogel, and the other was adsorbed on the surface of HAuNS nanoparticles in the PC_10_A hydrogel. Therefore, the DOX in PC_10_A/DOX/HAuNS hydrogels could be released step by step for chemotherapy. Compared with chemically cross-linked hydrogel, the release rate of DOX released from physical PC_10_A hydrogel was faster. This is probably because the release of DOX from PC_10_A hydrogel was contributed by the degradation of PC_10_A hydrogel and diffusion. The release rate of DOX from PC_10_A/DOX/HAuNS hydrogels in the acetate buffer of pH 5.0 was faster than that of in the PBS of pH 7.4, which was benefit for the drug release under acid microenvironment in tumor chemotherapy. Therefore, step-by-step release of DOX from PC_10_A/DOX/HAuNS hydrogels could provide a new strategy for long-term chemotherapy of tumors.

### Intratumoral retention experiment and drug distribution in vivo

**Fig. 6:**
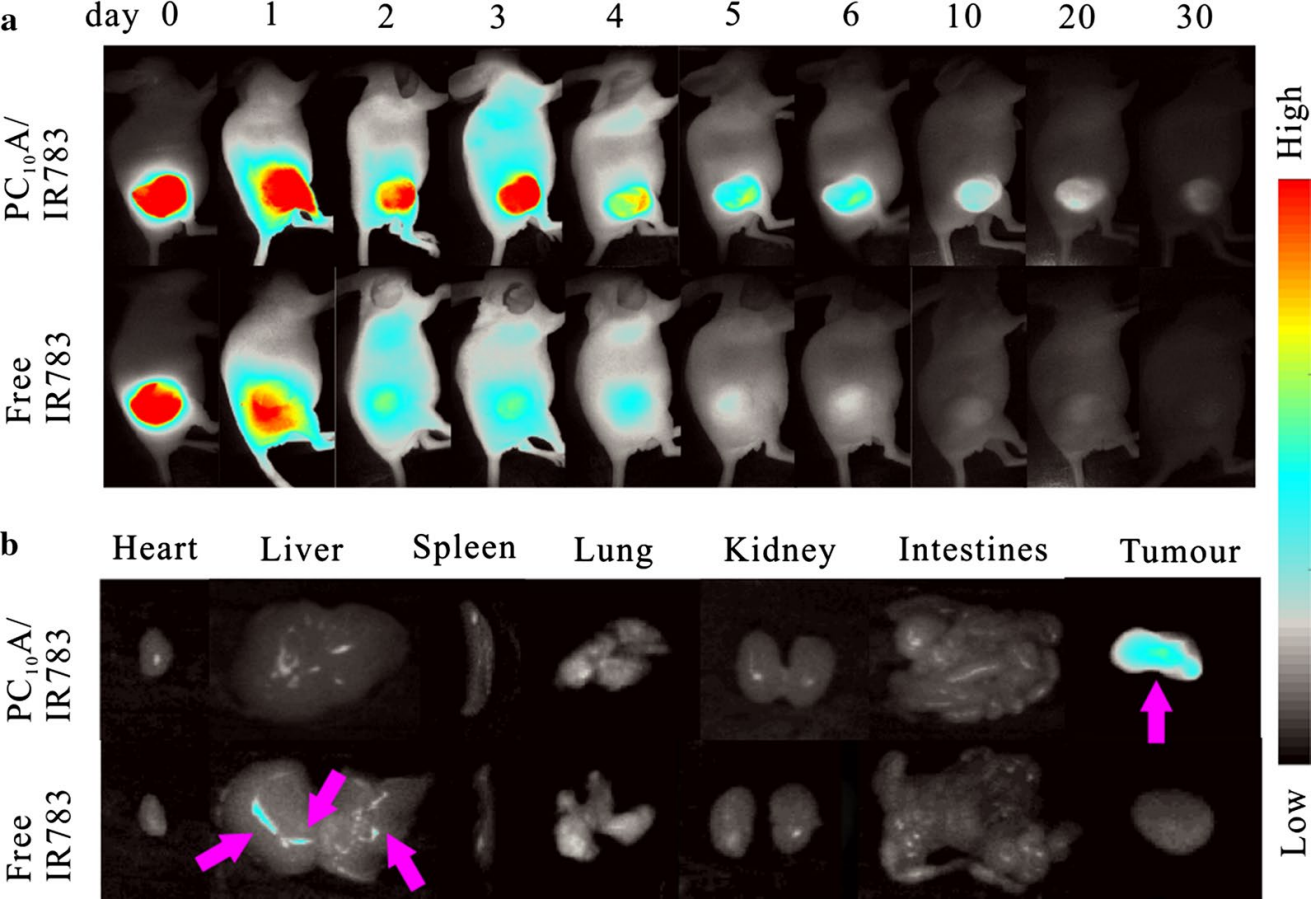
**a** Fluorescence imaging of the BALB/c nude mice injected with PC_10_A/IR783 hydrogel and IR783 solution (PC_10_A: 3% w/w, IR783: 40 μg mL^−1^) for different time intervals (day 0, 1, 2, 3, 4, 5, 6, 10, 20, and 30); **b** fluorescence imaging of the main organs (heart, liver, spleen, lung, and kidney) and tumors of the mice on day 6

To investigate the drug retardant capability of PC_10_A hydrogel, near infrared fluorescent dye IR783 encapsulated in the PC_10_A/IR783 hydrogel was utilized to monitor drug accumulation in tumor and distribution in major organs. Two groups of HepG2 tumor xenograft male BALB/c nude mice were set up as models. PC_10_A/IR783 hydrogel and free IR783 solution were respectively injected into the tumors with an equivalent IR783 dosage (40 μg mL^−1^). The release routine of IR783 was monitored by a self-built wide-field fluorescence imaging system (Fig. 6a). It is clearly seen that the fluorescence intensity decayed rapidly at the first day and almost vanished after injection on day 6 in the control group injected with free IR783 solution. However, no obvious change of fluorescent intensity in the PC_10_A/IR783 hydrogel positive group was observed on the third day, and a certain fluorescence signal of IR783 could still be seen on day 30. This result indicated that the release of IR783 of PC_10_A/IR783 hydrogel in tumors presented a sustained release process. To obtain a clearer insight of the distribution about IR783 in different organs (heart, liver, spleen, lung, and kidney) and tumors, another two groups of mice were sacrificed at day 6 after injection of PC_10_A/IR 783 hydrogel and free IR783 solution, respectively, and the fluorescence imaging of the main organs and tumors of the mice was measured. As shown in Fig. 6b, in the control group, almost no fluorescence signal was observed in the tumor and the main organs except in the liver. While IR783 in the PC_10_A hydrogel was mostly blocked at the site of the tumor. This result indicated that PC_10_A hydrogel could be used as a preeminent drug carrier for long term chemotherapy.

### Combination of photothermal therapy and chemotherapy in vitro and in vivo

**Fig. 7:**
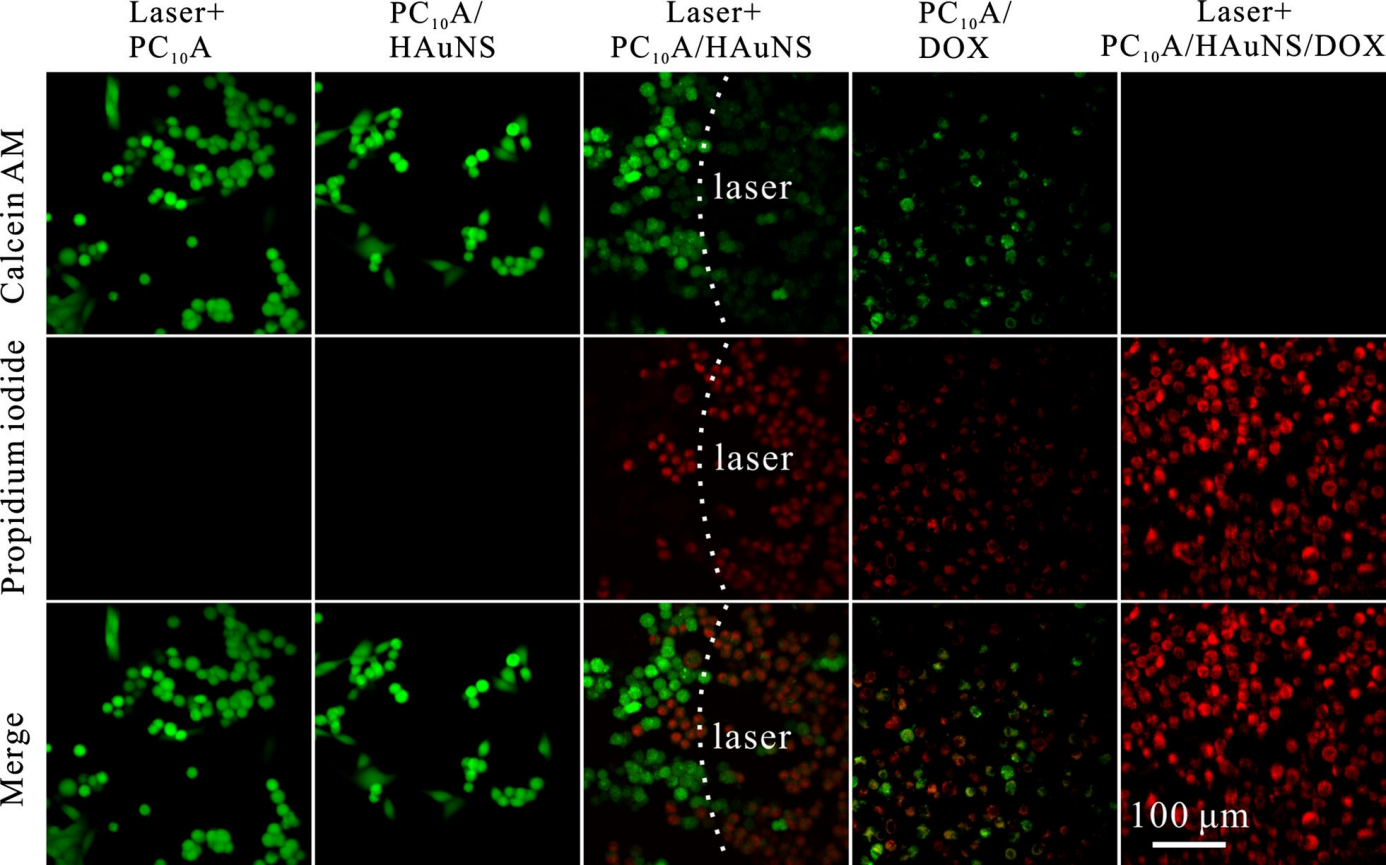
Confocal fluorescence images of HepG2 cells suffered with different treatments and stained with calcein AM/EthD-1 homodimer. The cells in PC_10_A hydrogel, PC_10_A/HAuNS hydrogel, and PC_10_A/DOX/HAuNS hydrogel were exposed under an 808 nm laser with a power density of 2.0 W cm^−2^ for 9 min (PC_10_A: 3% w/w; DOX: 0.8 mg mL^−1^, HAuNS: 20 μg mL^−1^)

The therapeutic efficacies of PC_10_A/HAuNS hydrogel, PC_10_A/DOX hydrogel, and PC_10_A/DOX/HAuNS hydrogel on HepG2 cells in vitro were evaluated. The therapeutic efficacy of blank PC_10_A hydrogel was used as a control. HepG2 cells embedded in PC_10_A hydrogel, PC_10_A/HAuNS hydrogel, and PC_10_A/DOX/HAuNS hydrogel were all irradiated with an 808 nm laser at the power density of 2.0 W cm^−2^ for 9 min. As shown in Fig. 7, cells in blank PC_10_A hydrogel and PC_10_A/HAuNS hydrogel without laser irradiation were all survived well, revealing that blank PC_10_A hydrogel and PC_10_A/HAuNS hydrogel without laser irradiation had no effect to HepG2 cells. Whereas more dead cells in PC_10_A/DOX hydrogel and PC_10_A/HAuNS hydrogel treated with laser irradiation were found. In addition, few living cells were observed in the PC_10_A/DOX/HAuNS hydrogel treated with laser irradiation. These results demonstrated that neither PC_10_A/HAuNS hydrogel treated with laser irradiation nor PC_10_A/DOX hydrogel could effectively kill all HepG2 cells, and the combination of chemotherapy and photothermal therapy could completely kill HepG2 cells in the PC_10_A/DOX/HAuNS hydrogel.

**Fig. 8:**
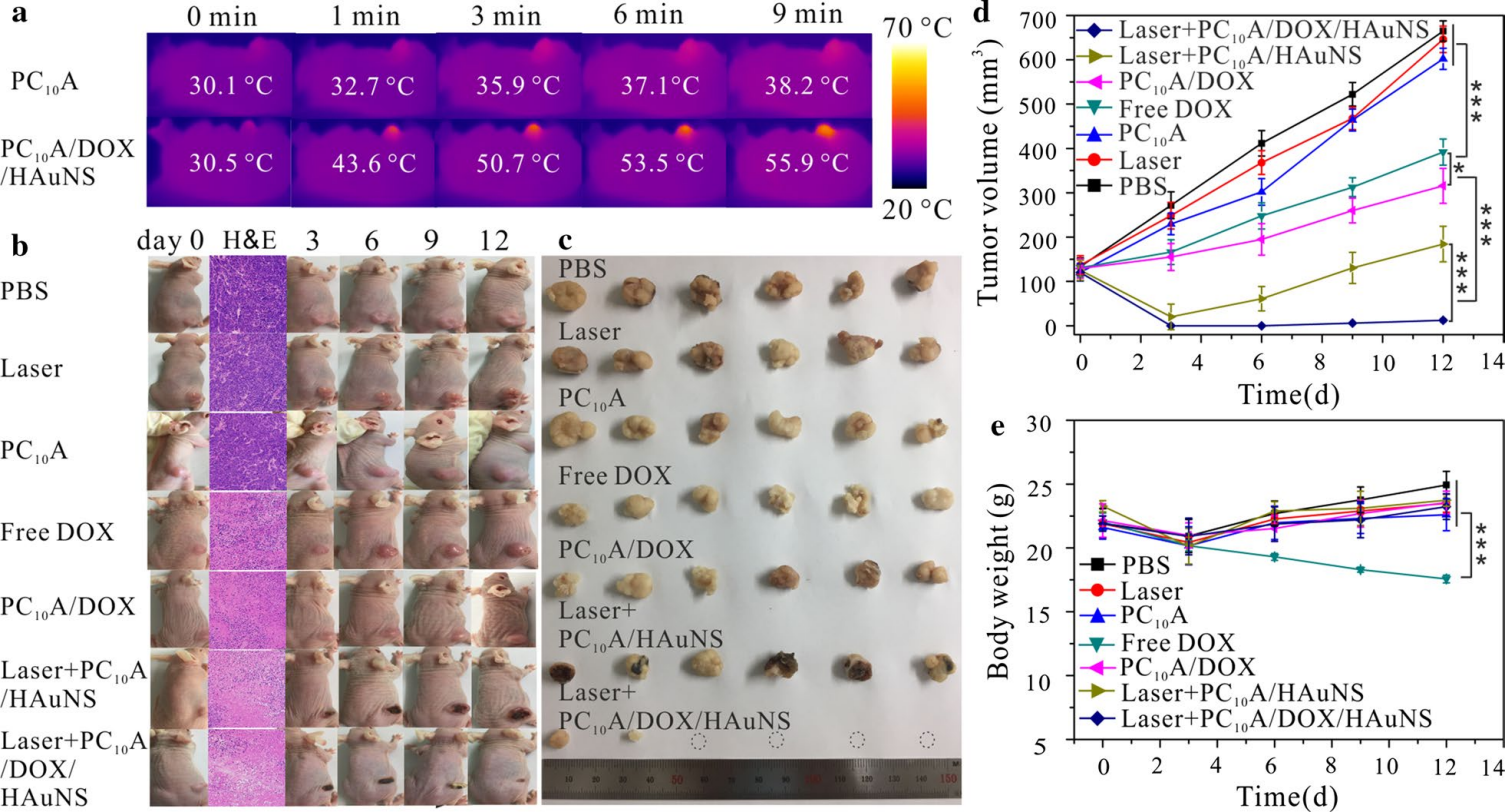
Anti-tumor efficiency of PC_10_A/DOX/HAuNS hydrogel in vivo. **a** Temperature changes of tumors after injected with PC_10_A hydrogel and PC_10_A/DOX/HAuNS hydrogel (100 μL) at different period of irradiation with an 808 nm laser at power density of 2.0 W cm^−2^; **b** photographs of the mice (H&E stained histological section of tumors after treatment for 1 day, the blue and pink indicated normal tissue cells and damaged tissue cells, respectively); **c** pictures of the tumors extracted from mice on day 12, **d** tumor volume and **e** body weight of the mice after treatment with PBS, laser irradiation, PC_10_A hydrogel, free DOX solution, PC_10_A/DOX hydrogel, PC_10_A/HAuNS hydrogel with laser, and PC_10_A/DOX/HAuNS hydrogel with laser (808 nm, 2.0 W cm^−2^, 9 min). n = 6, *p < 0.05, **p < 0.01, ***p < 0.001

To further study anti-tumor efficacy of PC_10_A/DOX/HAuNS hydrogel in vivo, the temperature changing of the HepG2 tumor injected with PC_10_A/DOX/HAuNS hydrogel under laser irradiation (λ = 808 nm, 2.0 W cm^−2^) was firstly measured. After exploring with the laser irradiation for 9 min, the temperature of the tumor reached up to 55.9 °C (Fig. 8a). While the temperature of the tumor injected with PBS only increased a little. These results suggested that the PC_10_A/DOX/HAuNS hydrogel still presented excellent photothermal effect in vivo. Therefore, the PC_10_A/DOX/HAuNS hydrogel is expected to be used as a photothermal agent for in vivo photothermal therapy of tumors.

Forty-two HepG2-tumor-bearing male mice were randomly divided into 7 groups: (1) PBS group, (2) laser irradiation alone group, (3) blank PC_10_A group, (4) free DOX group, (5) PC_10_A/DOX hydrogel group, (6) PC_10_A/HAuNS hydrogel group with laser irradiation, and (7) PC_10_A/DOX/HAuNS group with laser irradiation (n = 6). The in vivo antitumor efficacies of multifunctional PC_10_A/DOX/HAuNS hydrogel were tested through intratumoral injection. One mouse of each group was sacrificed for the tumor H&E stained histological section after treatment for 24 h to investigate the damage of tumor cells (Fig. 8b). Tumors injected with free DOX, PC_10_A/DOX hydrogel, PC_10_A/HAuNS hydrogel (laser+), and PC_10_A/DOX/HAuNS hydrogel (laser+) exhibited mass cell apoptosis. While no obvious damage were observed in PBS group, blank PC_10_A hydrogel group, and laser irradiation alone group, indicating that PC_10_A hydrogel and transient laser had no damage to tumor cells. After 12 days of treatment, the tumors in each group showed different growth trends. The sizes of tumors injected with PBS, PC_10_A hydrogel, and laser irradiation alone groups kept remarkable expanding during this course, suggesting that blank PC_10_A hydrogel and the laser irradiation alone could not suppress HepG2 tumor growth. Compared with the PBS control group, free DOX solution and PC_10_A/DOX hydrogel treatment groups delayed tumor growth. The sizes of tumors injected with PC_10_A/DOX hydrogel group were smaller than those of injected with free DOX solution. This is probably due to the sustained release of DOX from the PC_10_A/DOX hydrogel and the rapid cleaning of free DOX during blood circulation. Notably, tumors injected with PC_10_A/HAuNS hydrogel (laser+) and PC_10_A/DOX/HAuNS hydrogel (laser+) scarred rapidly after treatment, which may be due to the hyperthermia caused by laser irradiation (Fig. 8b). In the group of PC_10_A/HAuNS hydrogel (laser+), the sizes of tumors decreased sharply with time and then increased gradually. This is probably because pre-tumor inhibition was mainly due to the photothermal therapy of HAuNS in the hydrogel, and the recurrence was observed. This result indicated that photothermal therapy of HAuNS alone could not eliminate completely the tumor cells. As shown in Fig. 8c, d, the sizes of tumors treated with PC_10_A/DOX/HAuNS hydrogels (laser+) decreased with time, even 80% tumors disappeared completely, which has been attributed to the combination of photothermal therapy and sustained chemotherapy. At the same time, body weights were recorded during the cure period (Fig. 8e), all of them have no obvious difference except for the free DOX solution group, which may attribute to the adverse effect caused by the flourish diffusion of free DOX.

**Fig. 9:**
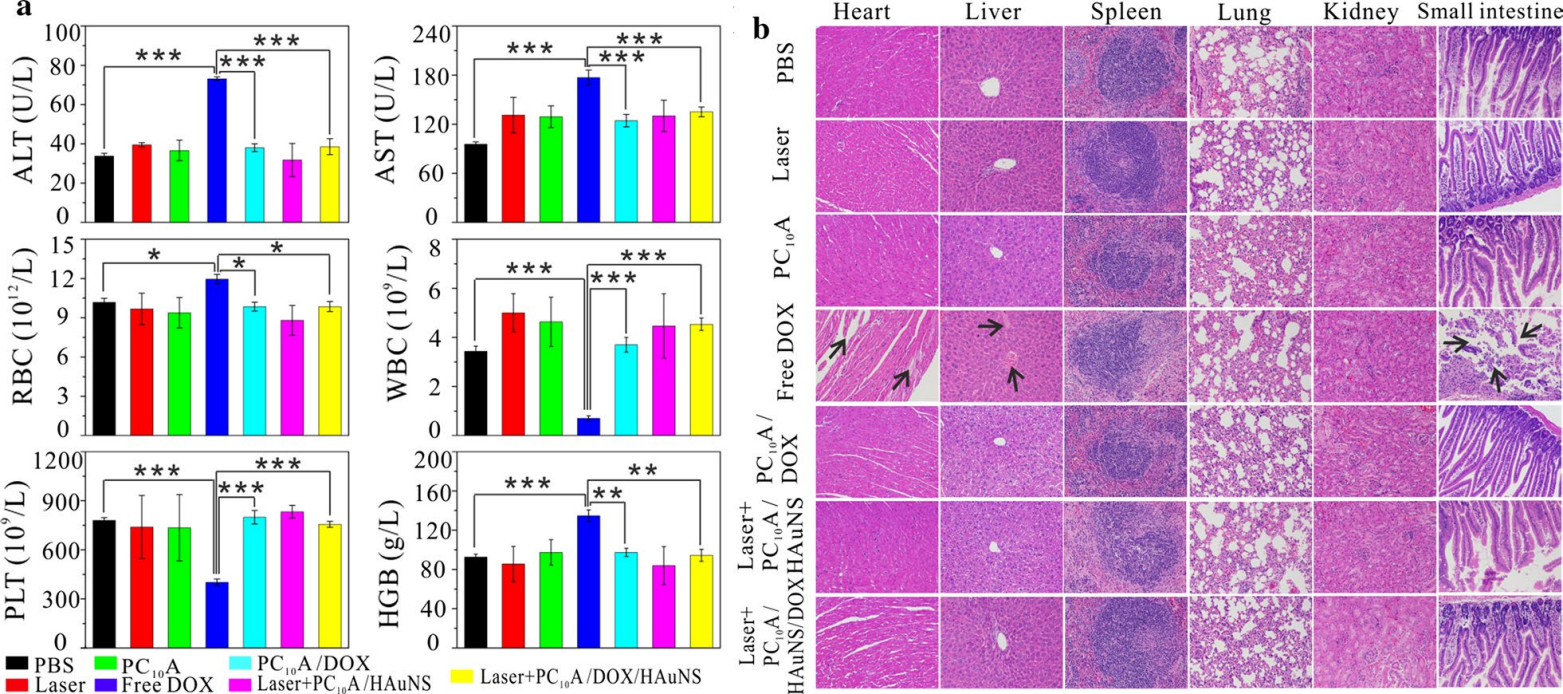
The blood assay and hematology analysis (**a**) and H&E staining results of the major organs (**b**) of the BALB/c mice after different treatments. The arrows indicated damaged tissue cells. n = 6, *p < 0.05, **p < 0.01, and ***p < 0.001

Mice were sacrificed to collect blood and the major organs after 12 days post-injection. The blood was used for serum biochemistry assays and routine hematology analysis, and organs were utilized for H&E stain (Fig. 9). Related serum biochemical assay (Fig. 9a) included concentration test of alanine aminotransferase (ALT) and aspartate aminotransferase (AST). The routine hematology analysis contained red blood cell count (RBC), white blood cell count (WBC), blood platelet count (PLT), and hemoglobin (HGB) test. The values of AST and ALT of the free DOX group were all beyond normal range, suggesting that the heart and liver of these mice probably suffered from serious dysfunction. The content of RBC and HGB of the free DOX group also increased obviously, which may be due to hemolysis caused by free DOX. All the other index of the free DOX group also showed significant variation compared with the control group, meaning that free DOX were toxic to the mice (n = 5, p < 0.05). The index values of other groups were all in normal section, indicating that there was no obvious blood toxicity. In addition, the histological tissue slices of major organs including heart, liver, spleen, lung, kidney and small intestine were also shown in Fig. 9b. Mice of the free DOX group exhibited some heart toxicity and led to severe inflammatory cells necrosis and gap enlarging. Simultaneously hyperemia bleeding and blood vessel congestion of the free DOX group appeared in hepatic sinus, and small intestinal villi distributed irregularly and seriously fell away. These results indicated that liver and small intestinal were also destructed partially. However, no obvious malformations were observed in other groups except for free DOX groups, suggesting that DOX encapsulated in PC_10_A hydrogel exhibited low systemic toxicity and were safe for in vivo applications.

### Long-term tumor therapy and the recurrence rate in vivo

**Fig. 10:**
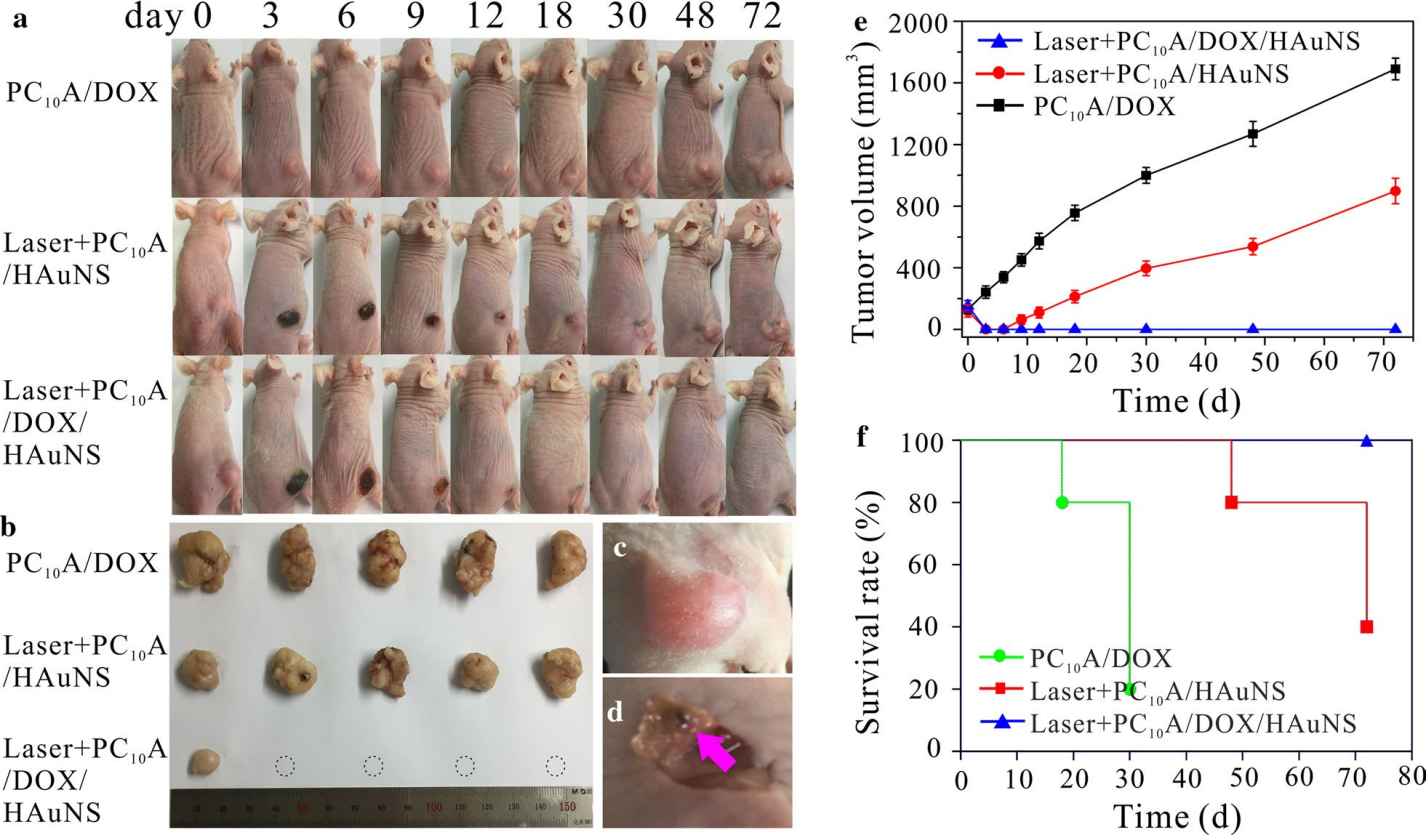
Long-term tumor therapy and the recurrence rate of HepG2 tumor bearing BALB/c mice. **a** Photographs of the mice after different treatment; **b** pictures of the tumors extracted from mice on day 72; Pictures of tumor before (**c**) and after (**d**) treatment with PC_10_A/DOX/HAuNS hydrogel on day 72 (There were still some PC_10_A/DOX/HAuNS hydrogels in the tumor site); Tumor volume (**e**) and survival rate (**f**) of the mice after treatment with PBS, laser irradiation, PC_10_A hydrogel, free DOX solution, PC_10_A/DOX hydrogel, PC_10_A/HAuNS hydrogel with laser, and PC_10_A/DOX/HAuNS hydrogel with laser (808 nm, 2.0 W cm^−2^, 9 min)

As we know, tumor recurrence is a tough hinder for the cancer therapy. To further evaluate the long-term therapy efficacies and recurrence rates of tumors, another three groups (n = 5) of mice were treated with PC_10_A/DOX hydrogel, PC_10_A/HAuNS hydrogel with laser irradiation, and PC_10_A/DOX/HAuNS hydrogel with laser irradiation, respectively. When the tumor volume was exceeded 1000 mm^3^, the mice were decided death [40]. Tumors were divided and took photographs at the end of the cure (Fig. 10a, b). As shown in the tumor volume curve and the survival curve (Fig. 10e, f), tumors injected with PC_10_A/DOX hydrogel kept growth over time and appeared mass mortality in 30 days. This result indicated that the chemotherapy alone presented a weak tumor inhibition efficacy. The volume of tumors injected with PC_10_A/HAuNS hydrogel (laser+) shrank quickly, but the volume of tumors began to gradually increase again on day 6. The survival rate descended radically in 70 days. These results showed that phototherapy alone could restrain the tumor in short time. In contrast, tumors treated with PC_10_A/DOX/HAuNS hydrogels (laser+) were suppressed perfectly. The recurrence rate and survived rate of mice after 72 days treatment with PC_10_A/DOX/HAuNS hydrogels (laser+) were 20% and 100%, respectively, which was superior to the single phototherapy or chemotherapy [41–43]. It is possible that a large number of tumor cells were first killed by photothermal treatment, and residual tumor cells were suppressed and eliminated by the DOX sustained releasing from PC_10_A/DOX/HAuNS hydrogels. Interestingly, on day 72, there were still some PC_10_A/DOX/HAuNS hydrogels in the tumor site of mice treated with PC_10_A/DOX/HAuNS hydrogels (laser+) were found (Fig. 10c, d), but the volumes were smaller than those of the pre-injection hydrogel. This result demonstrated that PC_10_A/DOX/HAuNS hydrogels could slowly degrade in vivo and sustained release of DOX for long-term chemotherapy. Therefore, this injectable PC_10_A/HAuNS/DOX hydrogel is beneficial to the development of combined photothermal therapy and long-term chemotherapy of tumors.

## Conclusion

In summary, we have developed a simple method to prepare the injectable PC_10_A/DOX/HAuNS hydrogel based on the polypeptide PC_10_A and HAuNS for chemo-photothermal therapy of HepG2 tumor. Hybrid PC_10_A/DOX/HAuNS nanoparticles were first prepared through self-assembled layer-by-layer, and multifunctional PC_10_A/DOX/HAuNS hydrogels were prepared through “dissolving” the hybrid PC_10_A/DOX/HAuNS nanoparticles in the PC_10_A hydrogel. The rheological properties of PC_10_A/DOX/HAuNS hydrogel were tuned by simply changing the concentrations of PC_10_A and HAuNS. Excitingly, this multifunctional PC_10_A/DOX/HAuNS hydrogel could pass 0.25 mm diameter needle without clogging. The results of photothermal effect showed that HAuNS in the PC_10_A/DOX/HAuNS hydrogel still possessed excellent photothermal efficiency and photothermal stability. The results of in vitro and in vivo toxicity showed that the PC_10_A/DOX/HAuNS hydrogel was non-toxic. Finally, the results of in vitro and in vivo treatment exhibited that the combined chemotherapy and photothermal therapy of PC_10_A/DOX/HAuNS hydrogels could significantly improve the therapeutic effect. Therefore, these results reported here provide a new strategy for sustained chemo-photothermal therapy.

## Supplementary information

**Additional file 1.** Additional figures.

## Data Availability

All data generated or analyzed during this study are included in this published article (and its additional files).

## Abbreviations

HAuNS: hollow gold nanoshells
DOX: doxorubicin Hydrochloride
IPTG: isopropyl-β-d-thiogalactoside
Ni-NTA: nickelnitrilotriacetic acid
HAuCl_4_·3H_2_O: gold chloride trihydrate
PVP: polyvinylpyrrolidone
calcein-AM: calcein acetoxymethyl ester
WBC: white blood cell
RBC: red blood cell
HGB: hemoglobin
PLT: platelet
ALT: alanine aminotransferase
AST: aspartate aminotransferase
PTT: photothermal therapy
G′: storage moduli
G″: loss moduli
