# Supplementary material for: Hollow gold nanoshells-incorporated injectable genetically engineered hydrogel for sustained chemo-photothermal therapy of tumor
Source: J Nanobiotechnology. 2019 Sep 17;17:99. doi: 10.1186/s12951-019-0532-9 (PMC6749695; doi:10.1186/s12951-019-0532-9)
Supplement: Supplementary file 1 — Additional file 1. Additional figures. [file 12951_2019_532_MOESM1_ESM.doc]

Additional Information

**Hollow gold nanoshells-incorporated injectable genetically engineered hydrogel for sustained chemo-photothermal therapy of tumor**

RuiMei Jin^1‡^, Jie Yang ^1‡^, DongHui Zhao^1^, XiaoLin Hou^1^, ChaoQing Li^1^, Wei Chen^1^, YuanDi Zhao^1, 2^, ZhongYuan Yin^3^, * and Bo Liu^1, 2^, *

^1^ Britton Chance Center for Biomedical Photonics at Wuhan National Laboratory for Optoelectronics - Hubei Bioinformatics & Molecular Imaging Key Laboratory, Collaborative Innovation Center for Biomedical Engineering, College of Life Science and Technology, Huazhong University of Science and Technology, Wuhan 430074, Hubei, P. R. China

^2^ Key Laboratory of Biomedical Photonics (HUST), Ministry of Education, Huazhong University of Science and Technology, Wuhan 430074, Hubei, P. R. China

^3^ Cancer center, Union Hospital, Tongji Medical College, Huazhong University of Science and Technology, Wuhan 430022, Hubei, P. R. China.

* Corresponding author.

Fax: (+) 86 27-8779-2202

E-mail address: yzyunion@163.com (Z.-Y. Yin)

E-mail address: [lbyang@mail.hust.edu.cn](mailto:lbyang@mail.hust.edu.cn) (B. Liu)

^1^ These authors contributed equally to this work


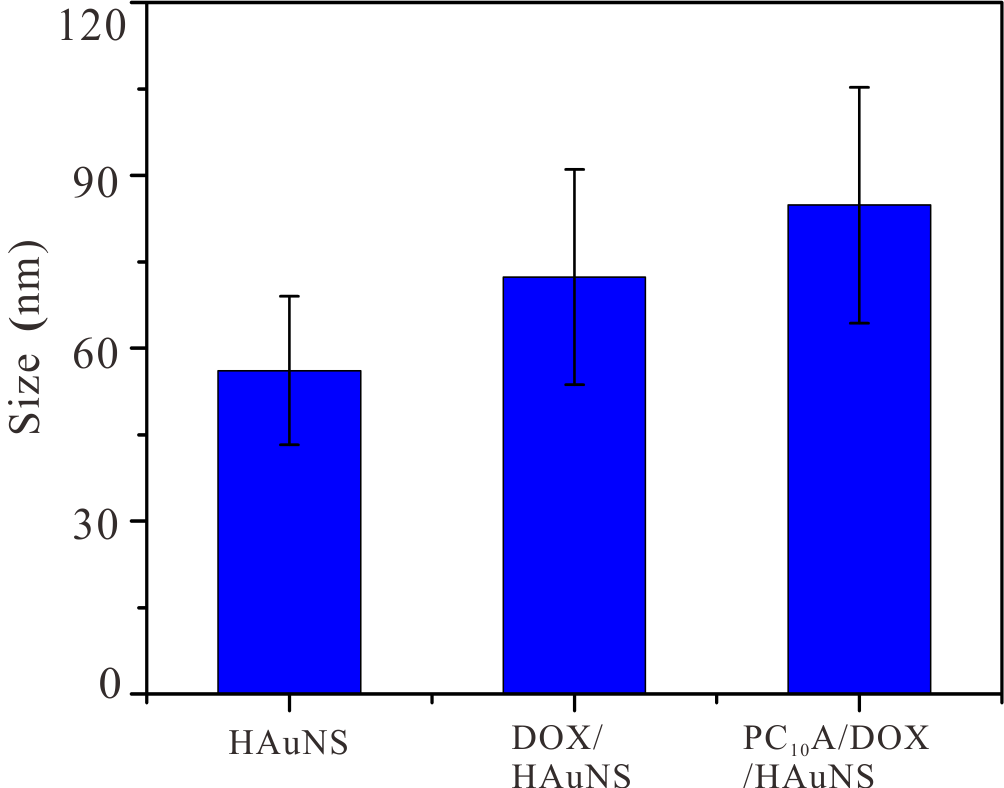


Fig. S1. The hydrodynamic sizes of HAuNS, DOX/HAuNS nanoparticles, and PC_10_A/DOX/HAuNS nanoparticles.


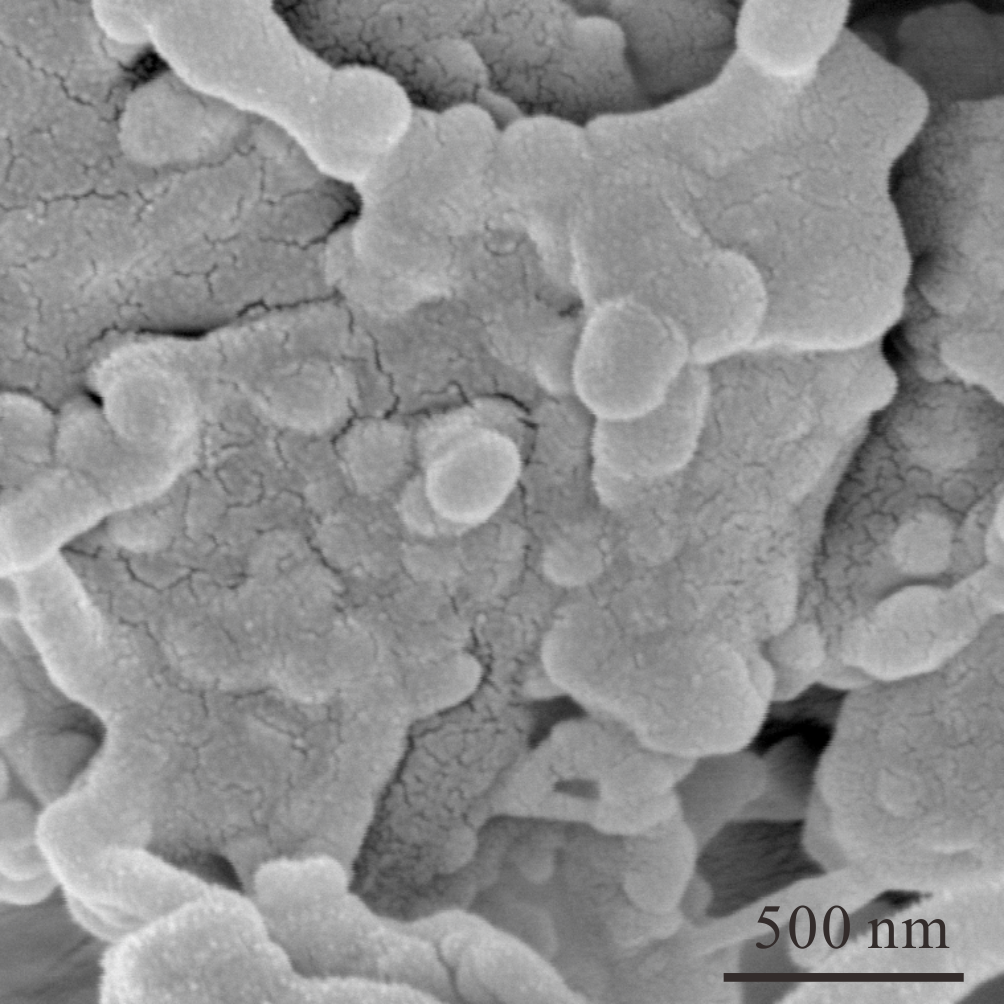


Fig. S2. High resolution SEM image of PC_10_A/DOX/HAuNS hydrogel.


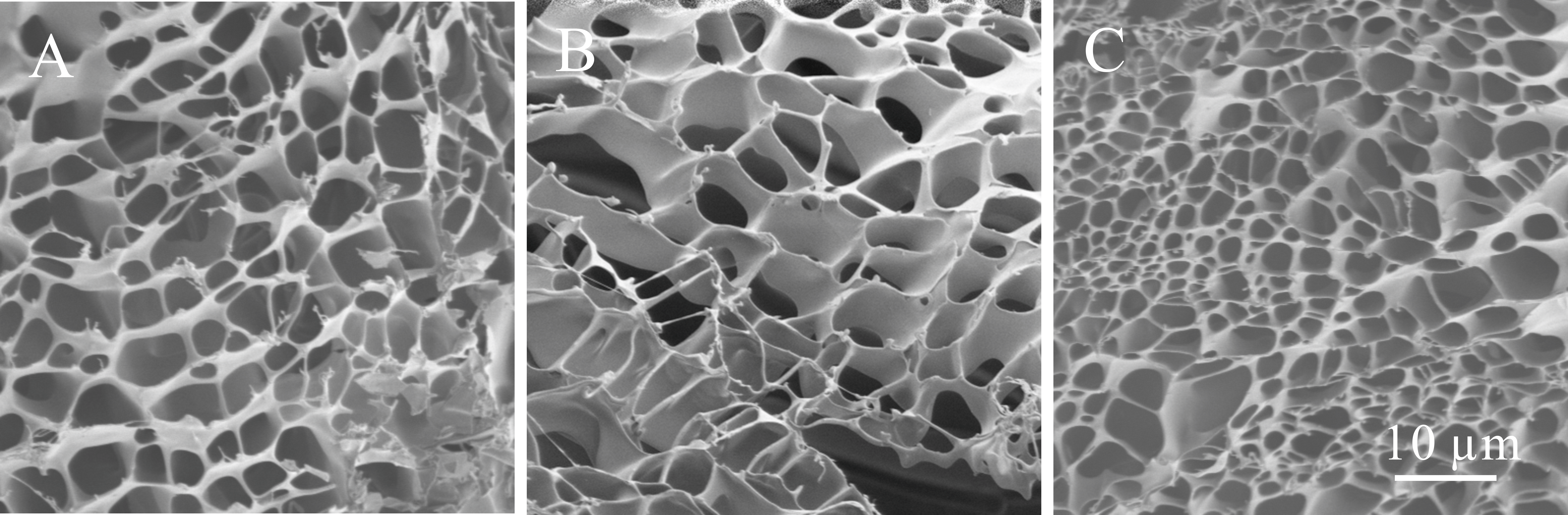


Fig. S3. SEM images of blank PC_10_A hydrogel (A), PC_10_A/DOX hydrogel (B), and PC_10_A/HAuNS hydrogel (C).


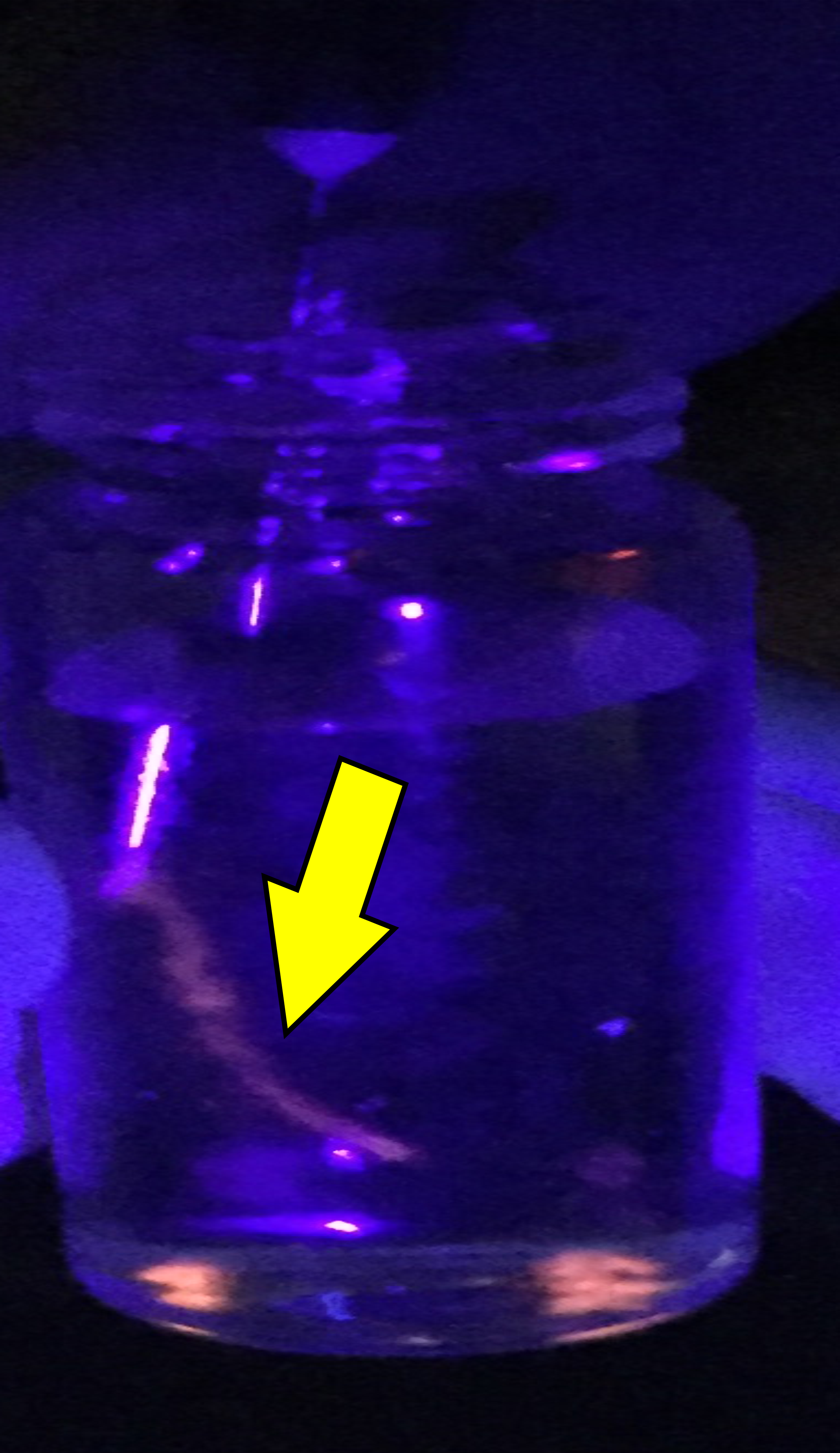


Fig. S4. PC_10_A/DOX/HAuNS hybrid hydrogel was transferred into a syringe and passed a 26-gauge needle.


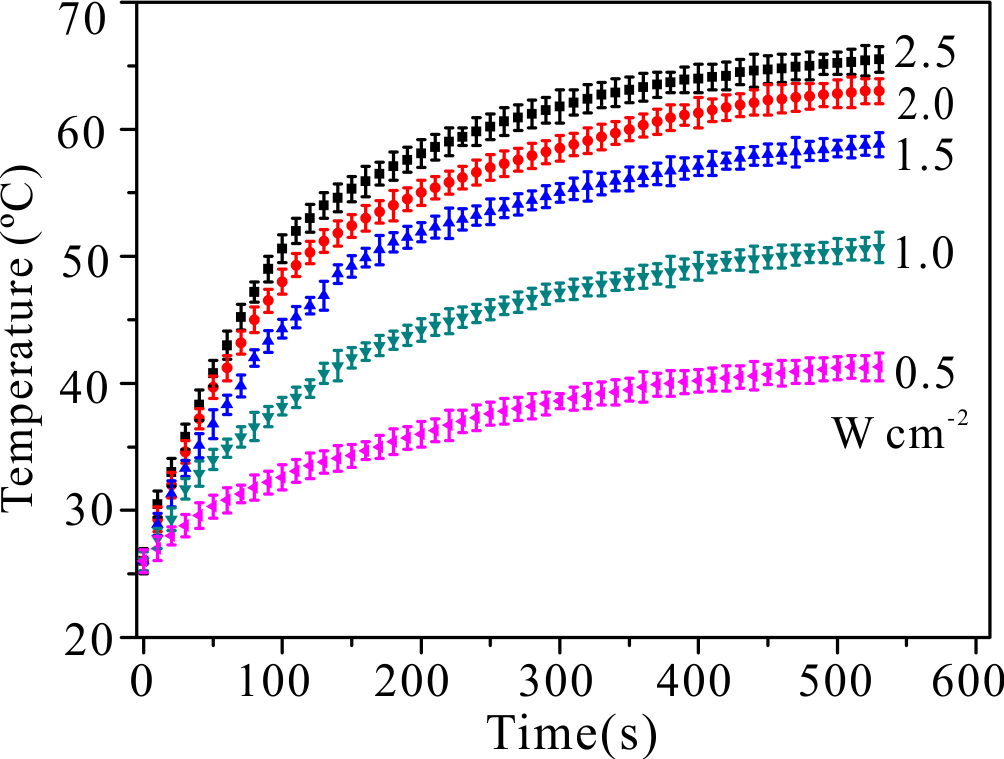


Fig. S5. Temperature changes of PC_10_A/DOX/HAuNS nanogels (PC_10_A: 0.1% w/w, DOX: 0.8 mg mL^-1^) irradiated with an 808 nm laser for 9 min under different power densities (0.5, 1.0, 1.5, 2.0, and 2.5 W cm^-2^). n = 3.


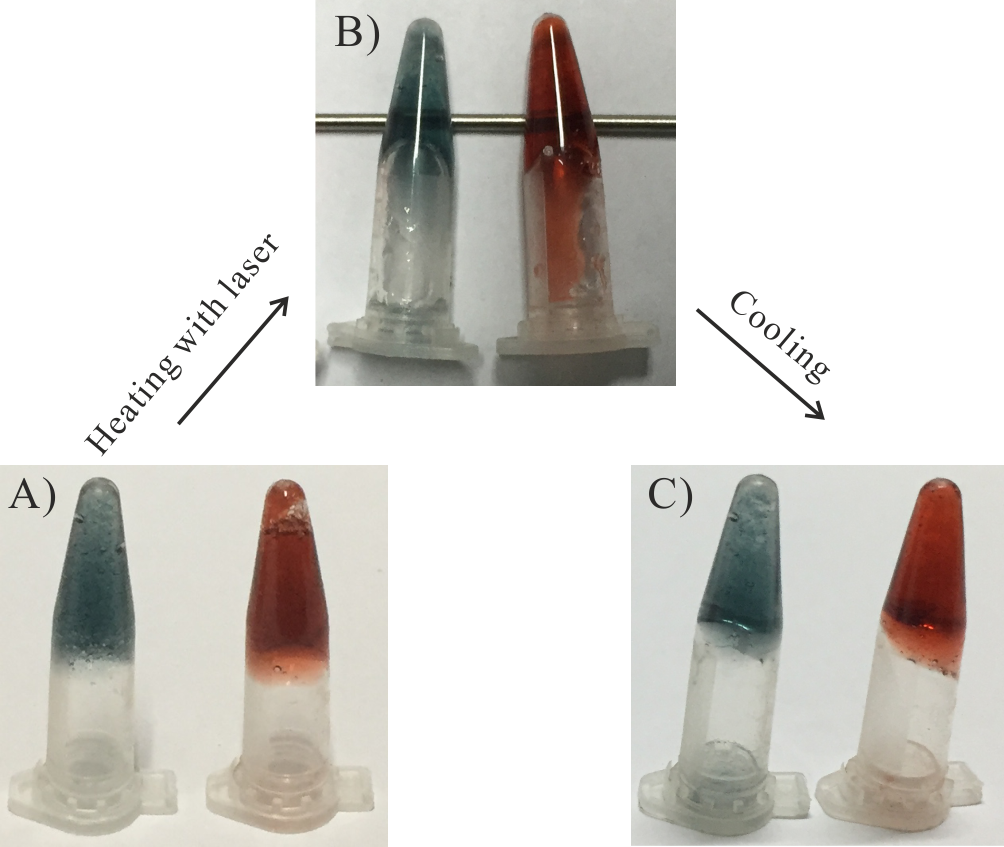


Fig. S6. (A) PC_10_A/HAuNS hydrogel and PC_10_A/DOX/HAuNS hydrogel (PC_10_A: 3% w/w, DOX: 0.8 mg mL^-1^, HAuNS: 20 μg mL^-1^) was exposed with an 808 nm laser at a power density of 2.0 W cm^-2^ for 9 min (B) and cooling down under room temperature (C).


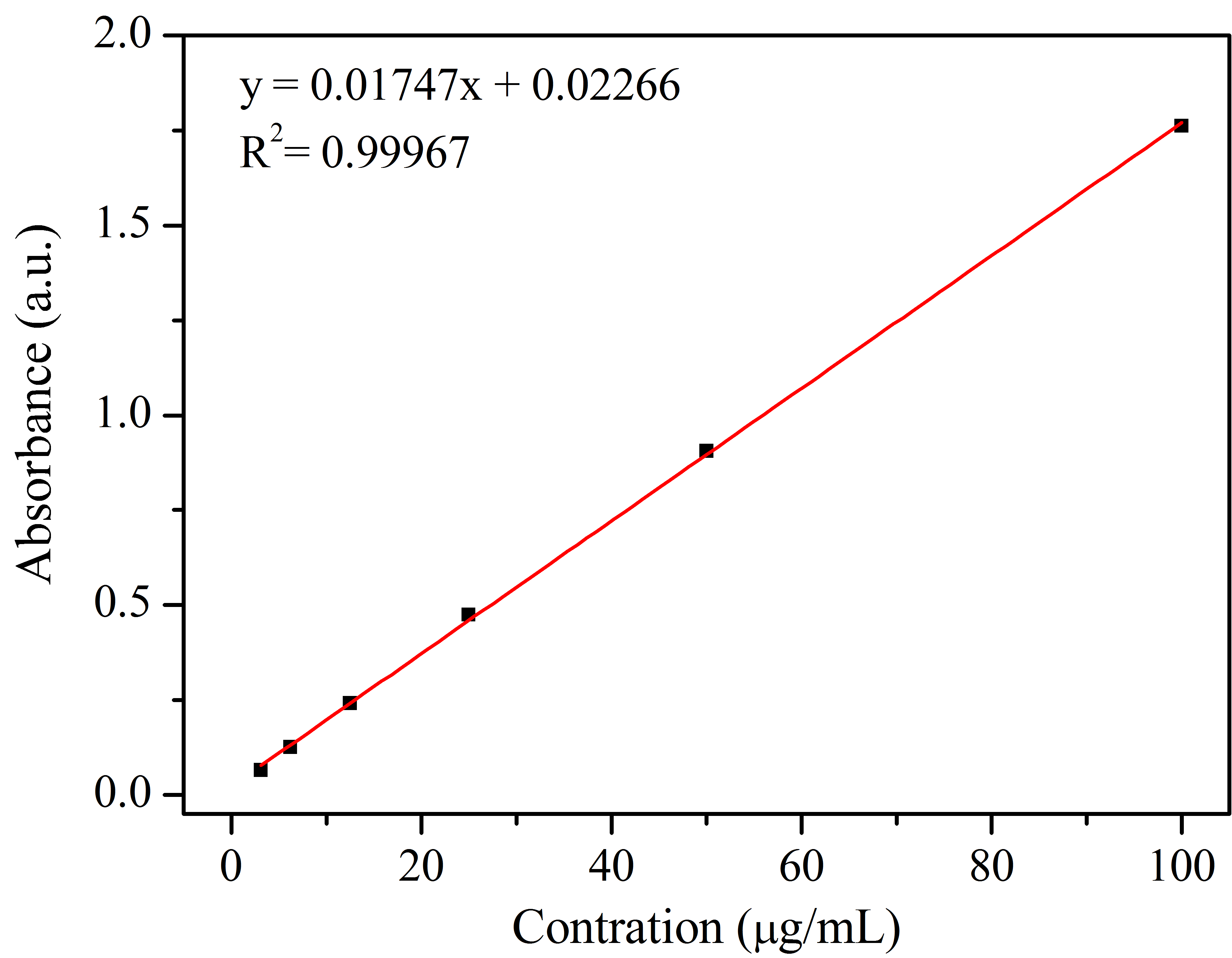


Fig. S7. Standard curve of DOX measured with absorption spectra at 480 nm.


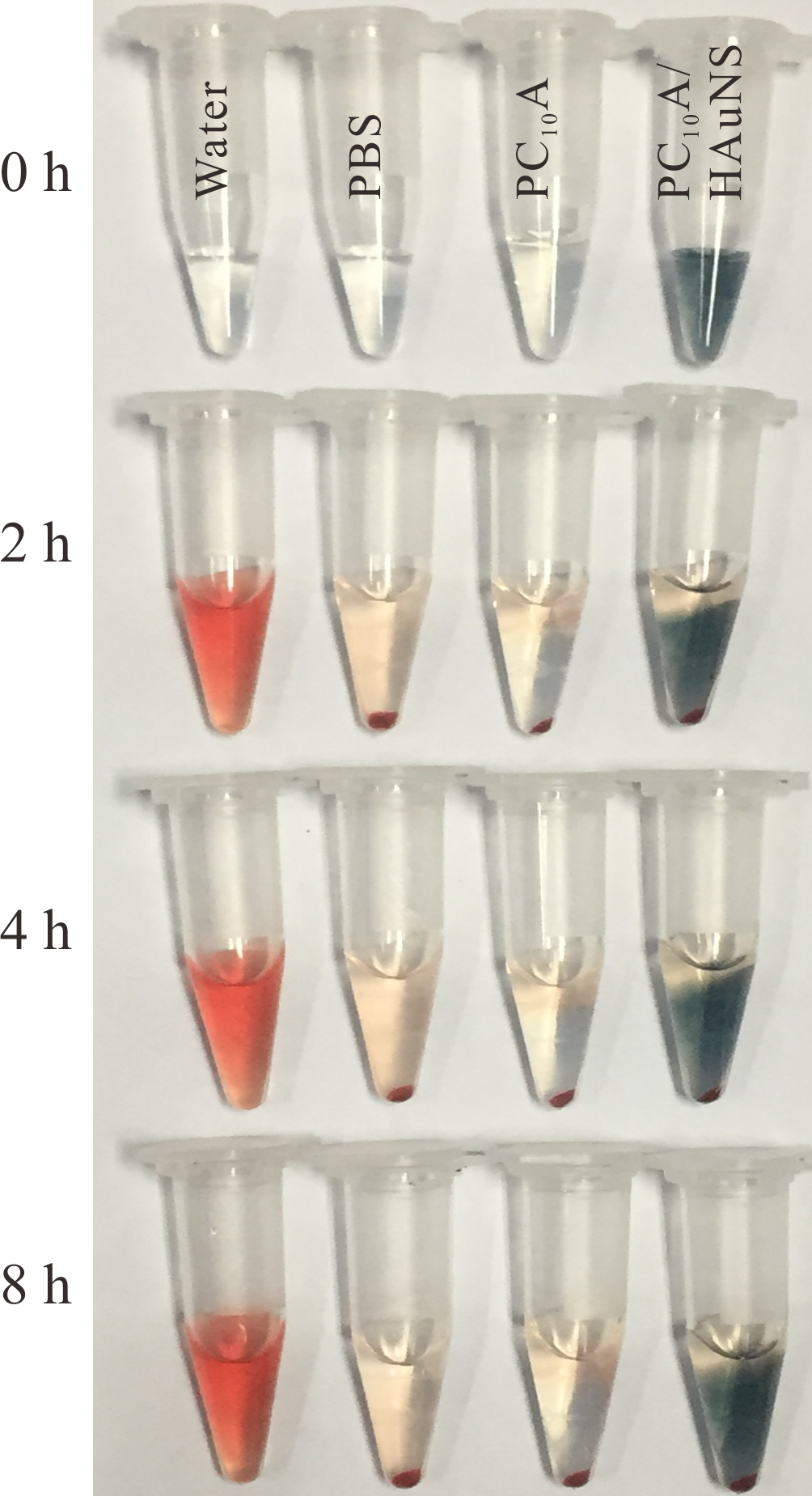


Fig. S8. Photographs of hemolysis ration of water, PBS, PC_10_A hydrogel, and PC_10_A/HAuNS hydrogel incubated with RBC for a series of time points (2, 4, and 8 h).
